# Cancer therapy and risk of congenital malformations in children fathered by men treated for testicular germ-cell cancer: A nationwide register study

**DOI:** 10.1371/journal.pmed.1002816

**Published:** 2019-06-04

**Authors:** Yahia Al-Jebari, Ingrid Glimelius, Carina Berglund Nord, Gabriella Cohn-Cedermark, Olof Ståhl, Torgrim Tandstad, Allan Jensen, Hege Sagstuen Haugnes, Gedske Daugaard, Lars Rylander, Aleksander Giwercman

**Affiliations:** 1 Molecular Reproductive Medicine, Department of Translational Medicine, Lund University, Malmö, Sweden; 2 Department of Medicine, Division of Clinical Epidemiology, Karolinska Institute, Stockholm, Sweden; 3 Department of Immunology, Genetics and Pathology, Uppsala University, Uppsala, Sweden; 4 Department of Oncology-Pathology, Karolinska Institute and Karolinska University Hospital, Stockholm, Sweden; 5 Department of Oncology, Skåne University Hospital, Lund, Sweden; 6 Department of Clinical and Molecular Medicine, Faculty of Medicine and Health Sciences, Norwegian University of Science and Technology, Trondheim, Norway; 7 The Cancer Clinic, St. Olav’s University Hospital, Trondheim, Norway; 8 Virus, Lifestyle and Genes, Danish Cancer Society Research Center, Copenhagen, Denmark; 9 Department of Oncology, University Hospital of North Norway, Tromsø, Norway; 10 Institute of Clinical Medicine, UiT The Arctic University of Norway, Tromsø, Norway; 11 Department of Oncology, Copenhagen University Hospital, Rigshospitalet, Copenhagen, Denmark; 12 Division of Occupational and Environmental Medicine, Lund University, Lund, Sweden; Vanderbilt University School of Medicine, UNITED STATES

## Abstract

**Background:**

Because of the potential mutagenic effects of chemo- and radiotherapy, there is concern regarding increased risk of congenital malformations (CMs) among children of fathers with cancer. Previous register studies indicate increased CM risk among children conceived after paternal cancer but lack data on oncological treatment. Increased CM risk was recently reported in children born before paternal cancer. This study aims to investigate whether anti-neoplastic treatment for testicular germ-cell cancer (TGCC) implies additional CM risk.

**Methods and findings:**

In this nationwide register study, all singletons born in Sweden 1994–2014 (*n* = 2,027,997) were included. Paternal TGCC diagnoses (*n* = 2,380), anti-neoplastic treatment, and offspring CMs were gathered from the Swedish Norwegian Testicular Cancer Group (SWENOTECA) and the Swedish Medical Birth Register. Children were grouped based on +/- paternal TGCC; treatment regimen: surveillance (*n* = 1,340), chemotherapy (*n* = 2,533), or radiotherapy (*n* = 360); and according to time of conception: pre- (*n* = 2,770) or post-treatment (*n* = 1,437). Odds ratios (ORs) for CMs were calculated using logistic regression with adjustment for parental ages, maternal body mass index (BMI), and maternal smoking. Children conceived before a specific treatment acted as reference for children conceived after the same treatment. Among children fathered by men with TGCC (*n* = 4,207), 184 had a CM. The risk of malformations was higher among children of fathers with TGCC compared with children fathered by men without TGCC (OR 1.28, 95% confidence interval [CI] 1.19–1.38, *p* = 0.001, 4.4% versus 3.5%). However, no additional risk increase was associated with oncological treatment when comparing post-treatment–to pretreatment-conceived children (chemotherapy, OR = 0.82, 95% CI 0.54–1.25, *p* = 0.37, 4.1% versus 4.6%; radiotherapy, OR = 1.01, 95% CI 0.25–4.12, *p* = 0.98, 3.2% versus 3.0%). Study limitations include lack of data on use of cryopreserved or donor sperm and on seminoma patients for the period 1995–2000—both tending to decrease the difference between the groups with TGCC and without TGCC. Furthermore, the power of analyses on chemotherapy intensity and radiotherapy was limited.

**Conclusions:**

No additional increased risk of CMs was observed in children of men with TGCC treated with radio- or chemotherapy. However, paternal TGCC per se was associated with modestly increased risk for offspring malformations. Clinically, this information can reassure concerned patients.

## Abstract 2

Yahia Al-Jebari and colleagues reveal no increased risk of congenital malformations in children of men treated for testicular germ cell cancer.

## Author summary

**Why was this study done?:**

- Chemotherapy and radiotherapy have both been shown to cause mutations and genetic damage in animal studies, leading to fears that men being treated with these therapies might be more prone to have children with genetic diseases and birth defects.
- This study investigates whether receiving treatment for testicular cancer, being the most common cancer among young men (in child-having ages), leads to a higher risk of fathering a child with a birth defect.

**What did the researchers do and find?:**

- By accessing data from Swedish national registries, we gathered data on more than 2 million newborns and their fathers. Among them, 2,380 fathers had testicular cancer and they had, in total, 4,207 children.
- When comparing the children conceived after the father received cancer treatment to the children born before treatment, we could not find any evidence showing that chemotherapy or radiotherapy leads to an increased risk of birth defects.
- We did see that there was, irrespectively of cancer treatment, a small risk increase associated with the fathers’ testicular cancer itself, meaning that men with testicular cancer have an approximately 30% higher relative risk of having a child with a birth defect (an increase from 3.5% to 4.4%).

**What do these findings mean?:**

- There was no risk increase due to cancer therapies.
- The risk increase detected was associated with testicular cancer. However, this risk increase is very small and relates to rare conditions.
- Because the risk increase is small, we therefore do not consider this cause for concern and wish this reassuring information to be passed on to patients.

## Introduction

Children fathered by men treated for cancer might be at higher risk for congenital malformations (CMs) due to the mutagenic effects of irradiation and cytotoxic drugs. Adverse effects of oncological treatments on germ cells have been described in animal [1,2] and human studies [3–5]. Furthermore, a Danish–Swedish population-based study has shown that children conceived after a father’s cancer diagnosis have a slight increase in prevalence of severe CM [6]. A possible pathway for the increased malformations risk might be detrimental genetic alterations of germline DNA by oncological treatment, resulting in more frequent CMs in children conceived after paternal oncological treatment.

There are indications that the excess risk of CMs for children born to fathers with cancer might be due to the malignancy per se rather than to the anticancer treatment. A Swedish population-based register study including 2.1 million children investigated the CM risk for children conceived prior to and after paternal cancer diagnosis and found those two groups to have an increased risk of malformations of about the same magnitude [7]. This study, when stratifying by cancer type, specifically showed that testicular cancer is one of the malignancies associated with increased CM risk in children conceived prior to paternal cancer diagnosis.

Testicular cancer is the most common cancer in young men, and approximately 97% of testicular cancers are derived from germ cells, so-called testicular germ-cell cancer (TGCC). TGCC has a 15-year survival of about 95% [8], and most patients will father children before or after the diagnosis. Among those TGCC patients who are childless at the time of cancer diagnosis, 77% express a wish for future fatherhood, despite some having anxieties about the potential detrimental health effects of cancer therapy on the health of their offspring [9].

Previous studies have lacked treatment data, making estimation of the possible additional effects of specific cancer therapies impossible. Opportunely, the Swedish Norwegian Testicular Cancer Group (SWENOTECA) registry includes treatment data on nonseminoma patients treated since 1995 and seminoma patients since 2000 for all Swedish and Norwegian TGCC patients. The main aim of this study was, by linking Swedish national registries to SWENOTECA, to investigate whether anti-neoplastic therapy implies any additional malformation risk in children fathered by men treated for TGCC. The secondary aim was to investigate whether TCGG per se is associated with risk of CM.

## Methods

### Study design and data sources

**Fig 1:**
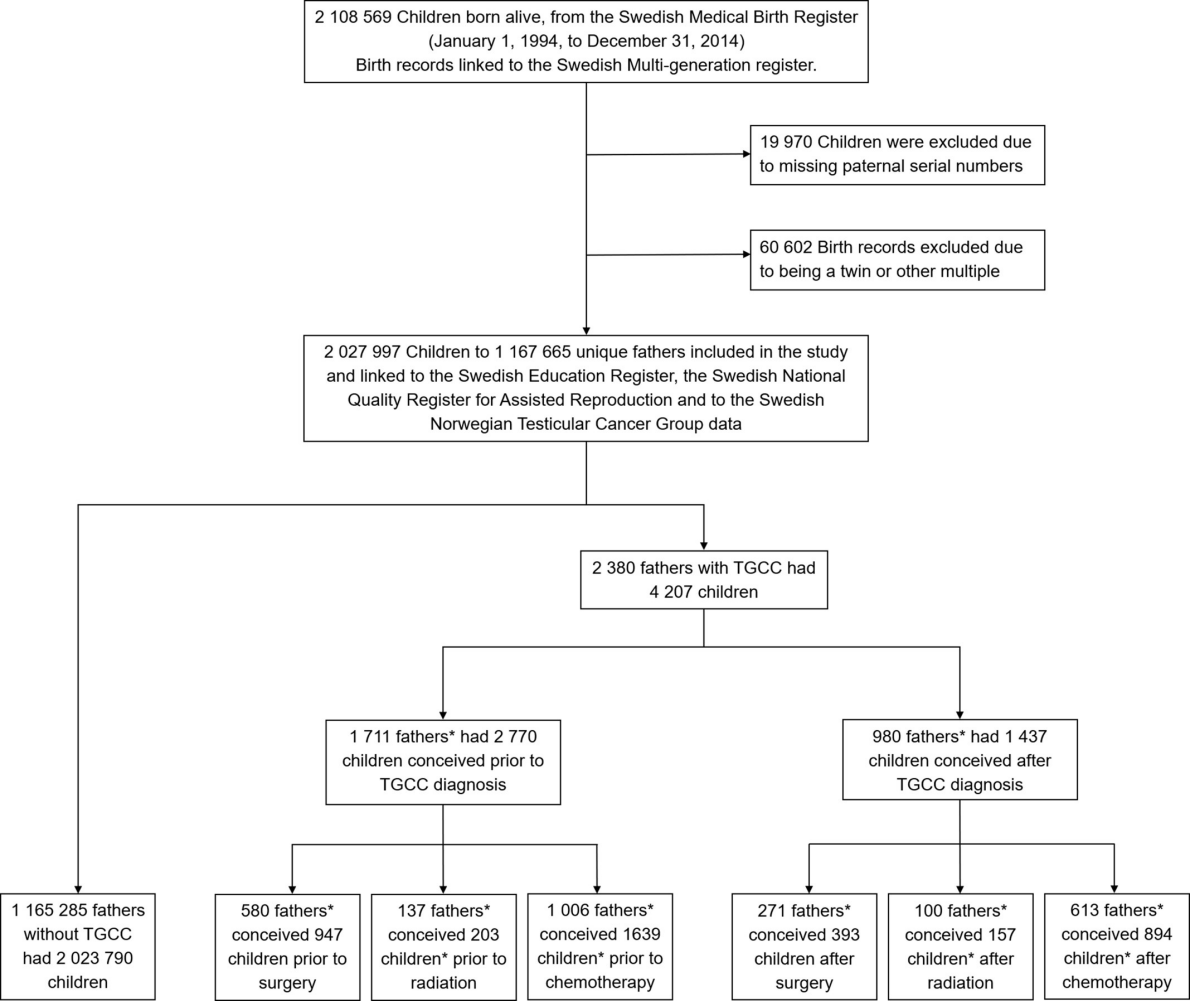
Definition of the study population, register linking, and subgrouping. *Numbers do not sum because there is overlap between groups. TGCC, testicular germ-cell cancer.

The cohort was defined as all newborns born alive and registered in the Swedish Medical Birth Register 1994–2014 (*n* = 2,108,569). Data from the Swedish Total Population Register and the Swedish Multigenerational Register allowed identification of their parents. Every individual in the cohort was given a unique serial number linked to their Swedish Personal Identity Number. These identification numbers were sent to the Swedish National Board of Health and Welfare, which supplied excerpts from national registries. To maintain anonymity, the Personal Identity Numbers were redacted by the Swedish National Board of Health and Welfare. Children with missing paternal serial numbers (*n* = 19,970) and twins and other multiples (*n* = 60,602) were excluded. After these exclusions, 2,027,997 singletons, 1,167,665 fathers, and 1,166,462 mothers remained in the cohort (Fig 1).

Perinatal and parental attributes for all children were obtained from Swedish Medical Birth Register, the Swedish National Quality Register for Assisted Reproduction, and the Swedish Register of Education. Paternal TGCC diagnoses and treatment data were retrieved from the Swedish part of SWENOTECA, which holds information on clinical stage, treatment, and follow-up for up to 10 years after diagnosis for nonseminoma patients since 1995 and seminoma patients since 2000. All Swedish cancer centers report to the SWENOTECA database, which is then cross-checked with the Swedish Cancer Registry every third month through the personal national registration numbers assigned to each resident in Sweden at birth or permanent residency. The completeness of the Swedish Cancer Registry is almost 100% [10]. The analysis plan is presented in S1 Text. The regional ethical boards of Lund and Stockholm approved this study (No: 2015/670 and 2016/2562-31/2).

### CMs

The classification of CM according to the International Classification of Diseases (ICD) codes has been described previously [7]. Abridgedly, the definitions used for all congenital abnormalities were ICD-9-SE 740–759 and ICD-10-SE Q00-Q99. Major malformations were classified following the coding guide of European Surveillance of Congenital Anomalies.

### Groups according to paternal oncological treatment

The grouping of the children was according to whether the child was conceived prior to or after the father’s TGCC diagnosis by using the gestational length for the child to estimate conception date. Both the children conceived prior to and after paternal TGCC diagnosis were subgrouped according to the fathers’ oncological treatment regimen into the following groups: chemotherapy, radiotherapy, and surgery only (surveillance). Chemotherapy regimens and number of cycles were defined according to the SWENOTECA cancer care protocols. Any dose of radiotherapy was counted as exposure.

### Statistical analyses

Risk estimates for congenital malformations were evaluated using a multivariable binary logistic regression model, yielding odds ratios (ORs) with 95% confidence intervals (CIs). The model was adjusted for the following covariates: maternal age at childbirth (continuous), paternal age at offspring birth (continuous), maternal body mass index (BMI) (categorical: <20, ≥20 to <25, ≥25 to <30, ≥30 to <35, ≥35 kg/m^2^), and self-reported maternal smoking at first prenatal visit (categorical: nonsmoker, 1–9 cigarettes per day, ≥10 cigarettes per day). These covariates were chosen because they have been previously shown to affect birth outcomes [11–13].

Multiple imputation by fully conditional specification was used to handle cases with missing data, creating 5 imputed data sets. All the variables and outcomes in the regression models were used as predictors to impute missing values for maternal weight, height, age at childbirth, and maternal smoking during pregnancy. For children born to fathers with TGCC and missing gestational length (*n* = 5), the median value (280 days) of the cohort was used. Other covariates and outcomes in the model did not have missing data.

Analyses investigating the effect of paternal anti-TGCC treatment were performed to ensure comparability between groups. The type of oncological treatment given relates to the subtype of TGCC. Therefore, in order to adjust for the potential effect of aggressiveness of the paternal disease, children born to fathers who would, after the child’s conception, be treated with chemotherapy acted as the reference for children conceived after the father was exposed to chemotherapy. Similarly, the children born to fathers who would, after the offspring’s conception, receive radiation were the reference for the children born after radiation.

Because detrimental effects of chemotherapy on the offspring of treated patients might only be apparent at high treatment doses, a subanalysis was conducted in which the children were stratified according to the number of cycles of chemotherapy (1–2; 3–4; 5+) given to the father and if the children were conceived before or after paternal diagnosis.

The children to fathers were split into two groups according to whether they were conceived before (reference group) or after the fathers’ TGCC diagnosis. These two groups were compared to see whether the children conceived after diagnosis had an increased risk of malformations due to any form of treatment.

It has been previously reported that children born to fathers with TGCC have an increased risk of birth defects, even when the child is conceived prior to paternal oncological treatment [7]. Therefore, all the children to fathers with TGCC were compared to the children of fathers without TGCC (reference) to evaluate whether there was a difference in risk for congenital malformations. Separate sensitivity analyses excluded children conceived by assisted reproduction techniques (ARTs) and children conceived to fathers with a cancer diagnosis other than TGCC.

All statistical analyses were performed by the first author using SPSS version 25 (IBM Corp, Armonk, NY, USA). Statistical analyses were two-sided. Risk estimates were pooled from multiple imputed values, with *p* < 0.05 considered statistically significant.

## Results

### Study population

Overall, 2,027,997 children were included in the study cohort (Fig 1). Of the total number included, 4,207 (0.2%) had fathers diagnosed with TGCC. Of these, 2,770 (65.8%) were conceived prior to and 1,437 (34.2%) were conceived after the TGCC diagnosis. The distribution of children according to time of paternal TGCC diagnosis showing other parental characteristics and birth outcomes is presented in Table 1.

**Table 1:** The distribution of children according to paternal TGCC diagnosis with parental characteristics and birth outcomes.

*Characteristic*	No paternal TGCC	Conceived prior to paternal TGCC diagnosis	Conceived after paternal TGCC diagnosis
Total no. of children	2,023,790	2,770	1,437
***Parental characteristics***			
Maternal age at offspring birth, years, mean (SD)	29.9 (5.1)	29.0 (4.9)	31.1 (4.6)
Maternal BMI at early pregnancy, kg/m^2^, mean (SD)	24.4 (4.4)	24.2 (4.2)	24.5 (4.6)
Paternal age at offspring birth, years, mean (SD)	32.8 (6.2)	30.9 (4.9)	33.8 (4.8)
Nonsmoking mothers early in pregnancy, no. (%)	1,826,916 (90.3)	2,466.6 (89.0)	1,372 (95.5)
Mothers smoking 1–9 cigarettes per day, no. (%)	139,791.2 (6.9)	210.8 (7.6)	47.2 (3.3)
Mothers smoking more than 10 cigarettes per day, no. (%)	57,082.8 (2.8)	92.6 (3.3)	17.8 (1.2)
***Mode of conception***			
Assisted	42,521 (2.1)	71 (2.6)	201 (14.0)
***Birth characteristics***			
*Sex*, *no*. *(%)*			
Male	1,040,460 (51.4)*	1,428 (51.6)	723 (50.3)
Female	983,324 (48.6)*	1,342 (48.4)	714 (49.7)
***CMs***			
All congenital abnormalities, no. (%)	69,920 (3.5)	125 (4.5)	59 (4.1)
Major congenital abnormalities, no. (%)	43,714 (2.2)	80 (2.9)	42 (2.9)

Values are pooled over 5 imputed data sets.

*Excluding 6 children for whom sex was missing.

**Abbreviations:** BMI, body mass index; CM, congenital malformation; no., number; SD, standard deviation; TGCC, testicular germ-cell cancer.

The most common treatment was chemotherapy, and the largest group of children were those fathered by men treated with chemotherapy (total *n* = 2,533, 60.2%, Table 2). The distribution of fathers according to treatment modality and intensity is given in Table 3.

**Table 2:** Parental and perinatal characteristics for groupings based on when conception occurred in relation to paternal treatment regimen.

*Characteristic*	*Paternal treatment regimen*					
	Surgery only		Chemotherapy		Radiotherapy	
	Conceived before	Conceived after	Conceived before	Conceived after	Conceived before	Conceived after
Total no. of children	947	393	1,639	894	203	157
*Mode of conception*						
Assisted	29 (3.1)	39 (9.9)	40 (2.4)	139 (15.5)	4 (2.0)	26 (16.6)
***Birth characteristics***						
*Sex*, *no*. *(%)*						
Male	504 (53.2)	196 (49.9)	838 (51.1)	444 (49.7)	98 (48.3)	86 (54.8)
Female	443 (46.8)	197 (50.1)	801 (48.9)	450 (50.3)	105 (51.7)	71 (45.2)
CMs						
All congenital abnormalities, no. (%)	45 (4.8)	18 (4.6)	75 (4.6)	37 (4.1)	6 (3.0)	5 (3.2)
Major congenital abnormalities, no. (%)	27 (2.9)	11 (2.8)	50 (3.1)	28 (3.1)	4 (2.0)	4 (2.5)

There were 19 children conceived to fathers prior to treatment with both chemotherapy and radiotherapy; among them, one had a major malformation. Similarly, 7 children were conceived after both treatment modalities, with one major malformation among them. **Abbreviations:** CM, congenital malformation; no., number.

**Table 3:** Distribution of fathers according to cancer and oncological treatment.

*Paternal TGCC*	Seminoma*	Nonseminoma^†^	All TGCC
Total no. of fathers (%)	1,308 (55.0)	1,072 (45.0)	2,380
Age at diagnosis, years, mean (SD)	37.3 (6.9)	31.9 (7.2)	34.9 (7.5)
***Treatment modality***			
Surgery only, no. of fathers (%)	477 (61.8)	295 (38.2)	772
Age at diagnosis, years, mean (SD)	37.8 (6.9)	33.0 (7.9)	35.9 (7.7)
***Chemotherapy***^‡^			
No. of fathers (%)	638 (45.2)	774 (54.4)	1,412
Age at diagnosis, years, mean (SD)	37.8 (6.9)	31.4 (6.8)	34.3 (7.6)
*Chemotherapy cycles*, *no*. *of fathers (%)*			
1–2	484 (56.3)	376 (43.7)	860
3–4	142 (29.2)	345 (70.8)	487
5+	1 (2.2)	44 (97.8)	45
Missing data	11 (55.0)	9 (45.0)	20
**Radiotherapy**^§^			
No. of fathers (%)	180 (98.9)	2 (1.1)	182
Age at diagnosis, years, mean (SD)	34.5 (5.8)	31.8 (4.2)	34.5 (5.8)
**Chemotherapy and radiotherapy**			
No. of fathers (%)	13 (92.9)	1 (7.1)	14
Average age at diagnosis, years (SD)	36.6 (8.9)	45.0 (-)	37.2 (8.8)

*Excluding patients also having nonseminoma (patients with mixed TGCC, seminoma and nonseminoma, are included in the nonseminoma group because they receive similar treatments under SWENOTECA cancer care protocols).

†Including patients also having seminoma.

‡Excluding patients also receiving radiotherapy.

§Excluding patients also receiving chemotherapy

**Abbreviations:** no., number; SD, standard deviation; SWENOTECA, the Swedish Norwegian Testicular Cancer Group; TGCC, testicular germ-cell cancer.

### CMs in relation to paternal TGCC treatment

When comparing the children conceived after particular anti-TGCC treatment regimen with the children conceived prior to the same regimen, there was no statistically significant increased risk after radiotherapy (all malformations: OR = 1.01, 95% CI = 0.25–4.12, *p* = 0.98, 3.2% versus 3.0%; major malformations: OR = 1.37, 95% CI = 0.27–7.05, *p* = 0.70, 2.5% versus 2.0%) or following chemotherapy (all malformations: OR = 0.82, 95% CI = 0.54–1.25, *p* = 0.37, 4.1% versus 4.6%; major malformations: OR = 1.01, 95% CI = 0.62–1.65, *p* = 0.97, 3.1% versus 3.1%).

The frequency of CMs according to the number of chemotherapy cycles the father was treated with is given in S1 Table. Although this analysis has low statistical power, in 5 of 6 subcategories, the OR of malformation risk was below 1 for the post-treatment children as compared to the prediagnosis group.

The children conceived after paternal TGCC diagnosis, as compared to those conceived before paternal TGCC, showed no risk difference either for all or for major malformations (OR = 0.88, 95% CI = 0.63–1.22, *p* = 0.43, 4.1% versus 4.5% and OR = 1.03, 95% CI = 0.69–1.53, *p* = 0.88, 2.9% versus 2.9%, respectively).

### CMs in children of TGCC men

**Fig 2:**
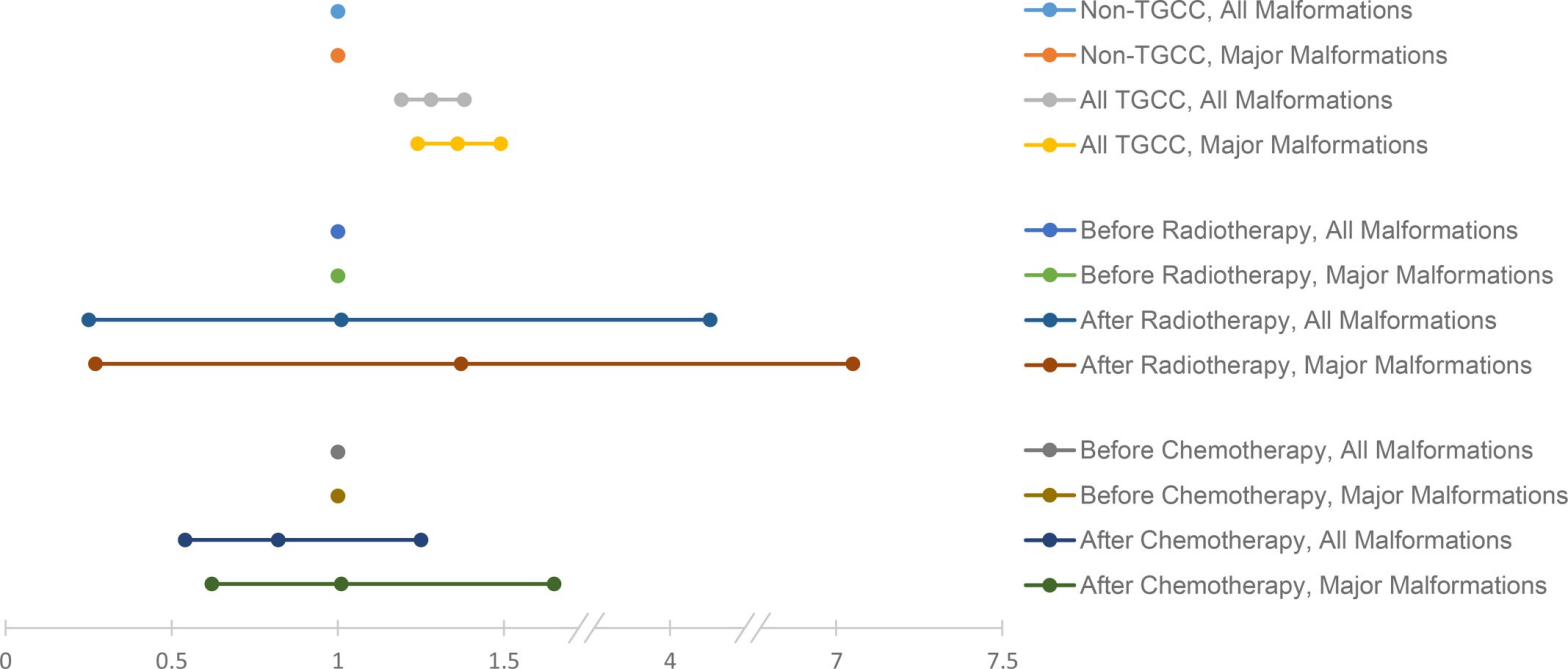
Forest plot of ORs and 95% CIs for risk of all and major malformations. Calculations were made according to the presence of TGCC or not and according to before or after treatment with radiotherapy or chemotherapy. CI, confidence interval; OR, odds ratio; TGCC, testicular germ-cell cancer.

Children to fathers with TGCC had a statistically significantly increased risk for both all and major CMs as compared to children born to fathers without TGCC (OR = 1.28, 95% CI = 1.19–1.38, *p* = 0.001, 4.4% versus 3.5% and OR = 1.36, 95% CI = 1.24–1.49, *p* < 0.001, 2.9% versus 2.2%, respectively; Fig 2).

In the sensitivity analyses excluding children conceived by assisted reproduction, we found negligible differences in risk estimates (all malformations: OR = 1.25, 95% CI = 1.16–1.36, *p* = 0.004, 4.3% versus 3.4%; major malformations: OR = 1.37, 95% CI = 1.25–1.51, *p* < 0.001, 2.9% versus 2.1%). Excluding the children of fathers with non-TGCC cancer from the reference group did not change the risk estimates (OR = 1.28, 95% CI = 1.19–1.38, *p* = 0.001, 4.4% versus 3.5% and OR = 1.36, 95% CI = 1.24–1.49, *p* < 0.001, 2.9% versus 2.2%).

## Discussion

In this study, we found that children of fathers with TGCC had an approximately 30% increased malformation risk as compared to children of fathers without TGCC. This modest increase applied to more severe forms of malformations, as well. However, when comparing children conceived after radio- or chemotherapy to those conceived prior to these potentially mutagenic treatments, there was no increased risk associated with either treatment. While very few in numbers, the same was true even for children conceived after more extensive paternal chemotherapy regimens. Altogether, our results indicate that although children born to fathers with TGCC have a significantly increased risk of all and major CMs, it is unlikely to be due to the effects of radio- or chemotherapy.

In 2011, we published a Danish–Swedish register study lacking information on paternal oncological treatment in which we observed that children born after paternal cancer diagnosis had a slight increase in CM rate [6]. This study indicated that the increase could be due to factors unrelated to cancer therapy because higher rates were also observed in children fathered by men with malignancies usually treated with surgery only. Supporting this thesis, our recent study showed that children conceived prior to a paternal cancer diagnosis, and therefore before treatment, were also more prone to have CM [7]. Few studies have addressed the issue of teratogenic risk following paternal oncological treatment, and none of the studies found an increased risk of CM [14–17]. Those studies, however, were not powered to detect small risk increases, such as those observed in this study. Parental exposure of mice to radiation and chemicals causes a variety of adverse effects (e.g., tumors, CMs, and embryonic deaths) in the progeny [18]. Direct exposure to ionizing radiation has been shown to increase the risk of childhood leukemia; however, paternal exposure has not been shown to affect the health of the offspring [19].

The association between the germ-cell–derived TGCC and CM risk in children is quite puzzling. In 2001, Skakkebaek and colleagues suggested that congenital genitourinary malformations, TGCC, and male infertility are caused by a common factor present during fetal life, being different manifestations of so-called testicular dysgenesis syndrome (TDS) [20]. According to the TDS hypothesis, the vast majority of testis tumors are derived from malignantly transformed precancerous germ-cell neoplasm in situ (GCNIS) cells, which in turn originate from mitotically arrested fetal gonocytes [21]. Although speculative, TDS could be the underlying syndrome linking paternal TGCC and offspring CMs. Because TDS is believed to be caused by a combination of environmental exposure and genetic factors in early fetal development, the same exposures/factors might lead to suboptimal development in the whole testes. Alternatively, the presence of GCNIS cells could itself be the cause of the increased risk of offspring malformations. It is unlikely that GCNIS cells undergo spermatogonial differentiation because of the genetic and morphological changes they exhibit. Instead, GCNIS cells, which occupy the same niche as germ cells, might influence the surrounding spermatogonal development through changes to the sensitive microenvironment within the seminiferous tubules. Indeed, infertility does often precede TGCC [22], and pretreatment TGCC patients have elevated levels of sperm DNA damage (higher DNA fragmentation index) [23], possibly due to perturbations of the physiologically unique meiotic DNA repair pathway occurring in spermatogonia. Conversely, infertility and sperm DNA damage has been observed preceding other types of malignancies as well. Therefore, other mechanisms might also be operating, aligning with our previous study showing that children conceived to men with other cancers, such as neurological malignancies, also had an increased risk of CM [7].

Increased levels of reactive oxygen species (ROSs) have also been suggested as a possible factor in pathogenesis of different malignancies. ROSs were shown to cause postmeiotic oxidative sperm DNA damage during a phase of germ-cell development when sperms lack DNA repair machinery [24]. Increased rates of these paternally derived DNA lesions, like single- and double-stranded breaks, have been shown to detrimentally affect embryonic development, rates of miscarriage, and pregnancy [25] and can ultimately lead to offspring with chromosomal aberrations [26]. Supporting this mechanism, our previous study indicated a 40% increase in chromosomal malformations among children born before paternal cancer [7].

Large genomic studies have shown that around 80% of de novo mutations occurring in the human population are derived from the paternal germline [27]. These de novo mutations among the offspring have been linked to CMs, schizophrenia, and autism [28–30]. Genomic studies have shown that the rate of de novo mutations that are passed on to offspring differs by more than 2-fold between fathers [27]. This indicates that some men experience more mutations in their germ cells and conceivably also in their somatic cells, possibly due to a systemic insufficiency of DNA repair. This genomic instability might itself predispose to malignancies.

Large genome-wide association studies have identified a multitude of risk loci associated with TGCC susceptibility. Interestingly, several of the loci identified support the TDS hypothesis with developmental arrest of fetal gonocytes because the loci are involved in transcriptional dysregulation. However, the same study suggests a multifactorial pathway might be at play because some risk loci were involved in defective microtubule function, which can lead to chromosomal instability [31].

Our findings have some potential clinical implications. First of all, although we find somewhat increased risk of CM in children fathered by men with TGCC, this increase is rather modest, and this reassuring information can be passed on to patients. Furthermore, our data do not seem to support the general assumption of increased risk of malformations in offspring of men treated with chemotherapy. Taken altogether, our results indicate that there is no cause for concern for men having undergone TGCC treatment regarding the health of their offspring. However, with 14% of the children born after TGCC treatment being conceived by ARTs, presumably due to high levels of infertility after gonadotoxic treatments, pretreatment sperm cryopreservation is still indicated since no tools for reliable prediction of post-treatment recovery of spermatogenesis are available.

The strength of this study is the utilization of large register data, which made it possible to estimate malformation risk in children conceived prior to as well as after TGCC diagnoses. Through linking the national registries with SWENOTECA, we had access to complete and detailed treatment data.

This study also had several limitations. Data on seminoma patients are lacking for the period 1995–2000. However, misclassification due to inclusion of children of those men in the control group should rather lead to diminishing the difference in malformation risk between TGCC offspring and the non-TGCC offspring. Furthermore, despite the use of national registries, the number of children with malformations fathered by men who received radiotherapy or very intensive chemotherapy treatment was still rather low. Therefore, the results for these subgroups should be taken with some caution. Another limitation is the lack of data regarding children born after insemination with cryopreserved or donor sperm. This was mitigated through the sensitivity analysis in which children born after assisted reproduction were excluded, which showed the same result.

To summarize, in this study comprising more than 2 million children, we found a slightly increased risk of CMs in children fathered by men diagnosed with TGCC. The magnitude of the risk was similar in those conceived prior to as well as post-paternal–cancer therapy, indicating no additional risk increase caused by radio- or chemotherapy.

## Supporting information

S1 STROBE Checklist STROBE, strengthening the reporting of observational studies in epidemiology. (DOCX)

S1 Table Frequency and risk estimates for all and major congenital malformations for children stratified according to the number of chemotherapy cycles the father has been treated with. (DOCX)

S2 Table Crude and adjusted risk estimates for all and major congenital malformations. (DOCX)

S3 Table Pooled risk estimates for all variables in the model comparing children conceived after paternal radiotherapy to children conceived before paternal radiotherapy. (DOCX)

S4 Table Pooled risk estimates for all variables in the model comparing children conceived after paternal chemotherapy to children conceived before paternal chemotherapy. (DOCX)

S5 Table Pooled risk estimates for all variables in the model comparing children conceived after paternal TGCC diagnosis as compared to those conceived before paternal TGCC. TGCC, testicular germ-cell cancer. (DOCX)

S6 Table Pooled risk estimates for all variables in the model comparing children conceived to fathers with TGCC as compared to those children born to fathers without TGCC. TGCC, testicular germ-cell cancer. (DOCX)

S7 Table Pooled risk estimates for all variables in the model comparing children conceived to fathers with TGCC as compared to those children born to fathers without TGCC, while excluding all children conceived through assisted reproductive techniques. TGCC, testicular germ-cell cancer. (DOCX)

S8 Table Pooled risk estimates for all variables in the model comparing children conceived to fathers with TGCC as compared to those children born to fathers without TGCC, while excluding all children to fathers that have any cancer other than TGCC. TGCC, testicular germ-cell cancer. (DOCX)

S9 Table Missing and imputed values in the original and the 5 imputed data sets. (DOCX)

S1 Text Analysis plan. (DOCX)

S2 Text Research protocol A. (DOCX)

S3 Text Research protocol B. (DOCX)

## Abbreviations

ART: assisted reproductive technique
BMI: body mass index
CI: confidence interval
CM: congenital malformation
GCNIS: germ-cell neoplasm in situ
ICD: International Classification of Diseases
OR: odds ratio
ROS: reactive oxygen species
SD: standard deviation
SWENOTECA: the Swedish Norwegian Testicular Cancer Group
TDS: testicular dysgenesis syndrome
TGCC: testicular germ-cell cancer
