# Supplementary material for: Cancer therapy and risk of congenital malformations in children fathered by men treated for testicular germ-cell cancer: A nationwide register study
Source: PLoS Med. 2019 Jun 4;16(6):e1002816. doi: 10.1371/journal.pmed.1002816 (PMC6548355; doi:10.1371/journal.pmed.1002816)
Supplement: S1 STROBE Checklist — (DOCX) [file pmed.1002816.s001.docx]

STROBE Statement—checklist of items that should be included in reports of observational studies

|  | Item No. | Recommendation | Section, Paragraph (§) | Relevant text from manuscript |
| --- | --- | --- | --- | --- |
| **Title and abstract** | 1 | (*a*) Indicate the study’s design with a commonly used term in the title or the abstract | Abstract, Methods and Findings, §1 | “In this nationwide register-study” |
|  |  | (*b*) Provide in the abstract an informative and balanced summary of what was done and what was found | Abstract, Methods and Findings, §1-2 | “Paternal TGCC diagnoses (n= 2380), anti-neoplastic treatment, and offspring CM were gathered from the Swedish Norwegian Testicular Cancer Group and the Swedish Medical Birth Register. Children were grouped based on: +/- paternal TGCC; treatment regimen: (surveillance (n=1340), chemotherapy (n=2533), or radiotherapy (n=360)); and according to time of conception: pre- (n= 2770) or post-treatment (n= 1437). Odds ratios (ORs) for CMs were calculated using logistic regression with adjustment for parental ages, maternal body mass index, and maternal smoking. Children conceived before a specific treatment acted as reference for children conceived after the same treatment.”  “Among children fathered by TGCC-men (n= 4207), 184 had a CM. The risk of malformations was higher among children of fathers with TGCC compared with children fathered by non-TGCC men (OR 1.28, 95% CI 1.19 - 1.38, p = 0.001, 4.4% vs. 3.5%). However, no additional risk increase was associated with oncological treatment (Chemotherapy, OR = 0.82, 95% CI 0.54 - 1.25, p= 0.37, 4.1% vs. 4.6%; Radiotherapy, OR = 1.01, 95% CI 0.25 - 4.12, p= 0.98, 3.2% vs.3.0%).” |
| Introduction | | | |  |
| Background/rationale | 2 | Explain the scientific background and rationale for the investigation being reported | Introduction, §1, 2, 4 | “Children fathered by men treated for cancer might be at higher risk for congenital malformations (CM) due to mutagenic effects of irradiation and cytotoxic drugs. Adverse effects of oncological treatments on germ cells has been described in animal [1, 2] and human studies [3–5]. [...] There are indications that the excess risk of congenital malformations for children born to fathers with cancer might be due to the malignancy per se rather than to the anti-cancer treatment. [...] Previous studies have lacked treatment data making estimation of the possible additional effects of specific cancer therapies impossible.” |
| Objectives | 3 | State specific objectives, including any prespecified hypotheses | Introduction, §4 | “The main aim of this study was, by linking Swedish national registries to SWENOTECA, to investigate whether anti-neoplastic therapy implies any additional malformation risk in children fathered by men treated for TGCC. The secondary aim was to investigate whether TCGG per se is associated with risk of CM.” |
| Methods | | | |  |
| Study design | 4 | Present key elements of study design early in the paper | Methods,  Study design and Data Sources, §1  Methods, Groups According to Paternal Oncological Treatment, §1 | “The cohort was defined as all newborns born alive and registered in the Swedish Medical Birth Register 1994-2014 (n=2 108 569). Data from Swedish Total Population Register and the Swedish Multigenerational Register allowed identification of their parents.”  “The grouping of the children was according to whether the child was conceived prior to or after the father’s TGCC diagnosis by using the gestational length for the child to estimate conception date. Both the children conceived prior to and after paternal TGCC diagnosis were sub-grouped according to the fathers’ oncological treatment regimen into the following groups: chemotherapy, radiotherapy, and surgery only (surveillance).” |
| Setting | 5 | Describe the setting, locations, and relevant dates, including periods of recruitment, exposure, follow-up, and data collection | Methods,  Study design and Data Sources, §1  Methods, Groups According to Paternal Oncological Treatment, §1 | “The cohort was defined as all newborns born alive and registered in the Swedish Medical Birth Register 1994-2014 (n=2 108 569). Data from Swedish Total Population Register and the Swedish Multigenerational Register allowed identification of their parents. [...] Perinatal and parental attributes for all children were obtained from Swedish Medical Birth Register, the Swedish National Quality Register for Assisted Reproduction, and the Swedish Register of Education. Paternal TGCC diagnoses and treatment data was retrieved from the Swedish part of SWENOTECA, which holds information on clinical stage, treatment, and follow-up for up to 10 years after diagnosis, for non-seminomas patients since 1995 and seminoma patients since 2000.”  “The grouping of the children was according to whether the child was conceived prior to or after the father’s TGCC diagnosis by using the gestational length for the child to estimate conception date. Both the children conceived prior to and after paternal TGCC diagnosis were sub-grouped according to the fathers’ oncological treatment regimen into the following groups: chemotherapy, radiotherapy, and surgery only (surveillance). Chemotherapy regimens and number of cycles were defined according to the SWENOTECA cancer care protocols. Any dose of radiotherapy was counted as exposure.” |
| Participants | 6 | (*a*) *Cohort study*—Give the eligibility criteria, and the sources and methods of selection of participants. Describe methods of follow-up  *Case-control study*—Give the eligibility criteria, and the sources and methods of case ascertainment and control selection. Give the rationale for the choice of cases and controls  *Cross-sectional study*—Give the eligibility criteria, and the sources and methods of selection of participants | Methods,  Study design and Data Sources, §1-2 | “The cohort was defined as all newborns born alive and registered in the Swedish Medical Birth Register 1994-2014 (n=2 108 569). [...] Children with missing paternal serial numbers (n=19 970), twins and other multiples (n=60 602) were excluded. After these exclusions, 2 027 997 singletons, 1 167 665 fathers, and 1 166 462 mothers remained in the cohort (Figure 1). [...] Perinatal and parental attributes for all children were obtained from Swedish Medical Birth Register, the Swedish National Quality Register for Assisted Reproduction, and the Swedish Register of Education. Paternal TGCC diagnoses and treatment data was retrieved from the Swedish part of SWENOTECA, which holds information on clinical stage, treatment, and follow-up for up to 10 years after diagnosis, for non-seminomas patients since 1995 and seminoma patients since 2000. “ |
|  |  | (*b*) *Cohort study*—For matched studies, give matching criteria and number of exposed and unexposed  *Case-control study*—For matched studies, give matching criteria and the number of controls per case |  | N/a |
| Variables | 7 | Clearly define all outcomes, exposures, predictors, potential confounders, and effect modifiers. Give diagnostic criteria, if applicable | Methods, Congenital Malformations, §1  Methods, Statistical Analyses, §1 | “The classification of CM according to the International Classification of Diseases codes has been described previously [7]. Abridgedly, the definition used for all congenital abnormalities was: ICD-9-SE 740-759 and ICD-10-SE Q00-Q99. Major malformations were classified following the coding guide of European Surveillance of Congenital Anomalies.”  “Risk estimates for congenital malformations were evaluated using a multivariable binary logistic regression model, yielding odds ratios (OR) with 95% confidence intervals (CI). The model was adjusted for the following covariates: maternal age at childbirth (continuous), paternal age at offspring birth (continuous), maternal BMI (categorical: <20, ≥20 to <25, ≥25 to <30, ≥30 to <35, ≥35 kg/m2), and self-reported maternal smoking at first prenatal visit (categorical: nonsmoker, 1-9 cigarettes per day, ≥10 cigarettes per day).” |
| Data sources/ measurement | 8* | For each variable of interest, give sources of data and details of methods of assessment (measurement). Describe comparability of assessment methods if there is more than one group | Methods,  Study design and Data Sources, §2  Methods, Groups According to Paternal Oncological Treatment, §1  Methods, Statistical Analyses, §3 | “Perinatal and parental attributes for all children were obtained from Swedish Medical Birth Register, the Swedish National Quality Register for Assisted Reproduction, and the Swedish Register of Education. Paternal TGCC diagnoses and treatment data was retrieved from the Swedish part of SWENOTECA, which holds information on clinical stage, treatment, and follow-up for up to 10 years after diagnosis, for non-seminomas patients since 1995 and seminoma patients since 2000.”  “The grouping of the children was according to whether the child was conceived prior to or after the father’s TGCC diagnosis by using the gestational length for the child to estimate conception date. Both the children conceived prior to and after paternal TGCC diagnosis were sub-grouped according to the fathers’ oncological treatment regimen into the following groups: chemotherapy, radiotherapy, and surgery only (surveillance).”  “Analyses investigating the effect of paternal anti-TGCC treatment were performed to ensure comparability between groups. The type of oncological treatment given relates to the subtype of TGCC. Therefore, in order to adjust for the potential effect of aggressiveness of the paternal disease, children born to fathers who would after the child’s conception be treated with chemotherapy acted as the reference for children conceived after the father was exposed to chemotherapy. Similarly, the children born to fathers who would after offspring’s conception receive radiation were the reference for the children born after radiation.” |
| Bias | 9 | Describe any efforts to address potential sources of bias | Methods, Statistical Analyses, §3 | “Analyses investigating the effect of paternal anti-TGCC treatment were performed to ensure comparability between groups. The type of oncological treatment given relates to the subtype of TGCC. Therefore, in order to adjust for the potential effect of aggressiveness of the paternal disease, children born to fathers who would after the child’s conception be treated with chemotherapy acted as the reference for children conceived after the father was exposed to chemotherapy. Similarly, the children born to fathers who would after offspring’s conception receive radiation were the reference for the children born after radiation.” |
| Study size | 10 | Explain how the study size was arrived at | Methods,  Study design and Data Sources, §1 | “The cohort was defined as all newborns born alive and registered in the Swedish Medical Birth Register 1994-2014 (n=2 108 569).” |

Continued on next page

| Quantitative variables | 11 | Explain how quantitative variables were handled in the analyses. If applicable, describe which groupings were chosen and why | | Methods, Statistical Analyses, §1 | “The model was adjusted for the following covariates: maternal age at childbirth (continuous), paternal age at offspring birth (continuous), maternal BMI (categorical: <20, ≥20 to <25, ≥25 to <30, ≥30 to <35, ≥35 kg/m2), and self-reported maternal smoking at first prenatal visit (categorical: nonsmoker, 1-9 cigarettes per day, ≥10 cigarettes per day). These covariates were chosen because they have been previously shown to affect birth outcomes11–13.” |  |
| --- | --- | --- | --- | --- | --- | --- |
| Statistical methods | 12 | (*a*) Describe all statistical methods, including those used to control for confounding | | Methods, Statistical Analyses, §1 | Risk estimates for congenital malformations were evaluated using a multivariable binary logistic regression model, yielding odds ratios (OR) with 95% confidence intervals (CI). The model was adjusted for the following covariates: maternal age at childbirth (continuous), paternal age at offspring birth (continuous), maternal BMI (categorical: <20, ≥20 to <25, ≥25 to <30, ≥30 to <35, ≥35 kg/m2), and self-reported maternal smoking at first prenatal visit (categorical: nonsmoker, 1-9 cigarettes per day, ≥10 cigarettes per day). |  |
|  |  | (*b*) Describe any methods used to examine subgroups and interactions | | Methods, Statistical Analyses, §3-6 | “Therefore, in order to adjust for the potential effect of aggressiveness of the paternal disease, children born to fathers who would after the child’s conception be treated with chemotherapy acted as the reference for children conceived after the father was exposed to chemotherapy. Similarly, the children born to fathers who would after offspring’s conception receive radiation were the reference for the children born after radiation. [...] As detrimental effects of chemotherapy on the offspring of treated patients might only be apparent at high treatment doses, a sub-analysis was conducted where the children were stratified according to the number of cycles of chemotherapy (1-2; 3-4; 5+) given to the father and if the children were conceived before or after paternal diagnosis[...]. The children to fathers were split into two groups according to if they were conceived before diagnosis (reference group) or after the fathers TGCC diagnosis. These two groups were compared to see if the children conceived after diagnosis had an increased risk of malformations due to any form of treatment.  It has been previously reported that children born to fathers with TGCC have an increased risk of birth defects, even when the child is conceived prior to paternal oncological treatment7. Therefore, all the children to fathers with TGCC were compared to the children of fathers without TGCC (reference) to evaluate if there was a difference in risk for congenital malformations. Separate sensitivity analyses excluded children conceived by assisted reproduction techniques (ART) and children conceived to fathers with a cancer diagnosis other than TGCC.” |  |
|  |  | (*c*) Explain how missing data were addressed | | Methods, Statistical Analyses, §2 | “Multiple imputation by fully conditional specification was used to handle cases with missing data, creating five imputed datasets. All the variables and outcomes in the regression models were used as predictors to impute missing values for maternal weight, height, age at childbirth, and maternal smoking during pregnancy. For children born to fathers with TGCC and missing gestational length (n=5), the median value (280 days) of the cohort was used. Other covariates and outcomes in the model did not have missing data.” |  |
|  |  | (*d*) *Cohort study*—If applicable, explain how loss to follow-up was addressed  *Case-control study*—If applicable, explain how matching of cases and controls was addressed  *Cross-sectional study*—If applicable, describe analytical methods taking account of sampling strategy | |  | N/a due to use of national register data |  |
|  |  | (*e*) Describe any sensitivity analyses | | Methods, Statistical Analyses, §6 | “Separate sensitivity analyses excluded children conceived by assisted reproduction techniques (ART) and children conceived to fathers with a cancer diagnosis other than TGCC.” |  |
|  | | | Results | | | |
| Participants | 13* | (a) Report numbers of individuals at each stage of study—eg numbers potentially eligible, examined for eligibility, confirmed eligible, included in the study, completing follow-up, and analysed | | Results, Study Population, §1-2 | Overall, 2 027 997 children were included in the study cohort (Figure 1). Of the total number included, 4 207 (0.2%) had fathers diagnosed with TGCC. Of these, 2 770 (65.8%) conceived prior to and 1437 (34.2%) were conceived after the TGCC diagnosis. The distribution of children according to time of paternal TGCC diagnosis showing other parental characteristics and birth outcomes is presented in Table 1. [...] The most common treatment was chemotherapy, and the largest group of children were those fathered by men treated with chemotherapy (total n=2 533, 60.2%, Table 2). The distribution of fathers according to treatment modality and intensity is given in Table 3” |  |
|  |  | (b) Give reasons for non-participation at each stage | |  | N/a |  |
|  |  | (c) Consider use of a flow diagram | | Figure 1 | Figure 1 |  |
| Descriptive data | 14* | (a) Give characteristics of study participants (eg demographic, clinical, social) and information on exposures and potential confounders | | Table 1 | Table 1. The distribution of children according to paternal TGCC diagnosis with parental characteristics and birth outcomes. |  |
|  |  | (b) Indicate number of participants with missing data for each variable of interest | | Table 3 | Table 3. Distribution of fathers according to cancer and oncological treatment, Missing data |  |
|  |  | (c) *Cohort study*—Summarise follow-up time (eg, average and total amount) | |  | N/a as outcome (congenital malformations) is diagnosed at birth |  |
| Outcome data | 15* | *Cohort study*—Report numbers of outcome events or summary measures over time | | Results  Study Population, §1 | “Of the total number included, 4 207 (0.2%) had fathers diagnosed with TGCC. Of these, 2 770 (65.8%) conceived prior to and 1437 (34.2%) were conceived after the TGCC diagnosis. The distribution of children according to time of paternal TGCC diagnosis showing other parental characteristics and birth outcomes is presented in Table 1.” |  |
|  |  | *Case-control study—*Report numbers in each exposure category, or summary measures of exposure | |  | N/a |  |
|  |  | *Cross-sectional study—*Report numbers of outcome events or summary measures | |  | N/a |  |
| Main results | 16 | (*a*) Give unadjusted estimates and, if applicable, confounder-adjusted estimates and their precision (eg, 95% confidence interval). Make clear which confounders were adjusted for and why they were included | | Supplementary Table S2  Methods, Statistical Analyses, §1 | Supplementary Table S2. Crude and adjusted risk estimates for all and major congenital malformations  “Risk estimates for congenital malformations were evaluated using a multivariable binary logistic regression model, yielding odds ratios (OR) with 95% confidence intervals (CI). The model was adjusted for the following covariates: maternal age at childbirth (continuous), paternal age at offspring birth (continuous), maternal BMI (categorical: <20, ≥20 to <25, ≥25 to <30, ≥30 to <35, ≥35 kg/m2), and self-reported maternal smoking at first prenatal visit (categorical: nonsmoker, 1-9 cigarettes per day, ≥10 cigarettes per day).” |  |
|  |  | (*b*) Report category boundaries when continuous variables were categorized | | Methods, Statistical Analyses, §1 | “The model was adjusted for the following covariates: maternal age at childbirth (continuous), paternal age at offspring birth (continuous), maternal BMI (categorical: <20, ≥20 to <25, ≥25 to <30, ≥30 to <35, ≥35 kg/m2), and self-reported maternal smoking at first prenatal visit (categorical: nonsmoker, 1-9 cigarettes per day, ≥10 cigarettes per day).” |  |
|  |  | (*c*) If relevant, consider translating estimates of relative risk into absolute risk for a meaningful time period | |  | N/a |  |

| Other analyses | 17 | Report other analyses done—eg analyses of subgroups and interactions, and sensitivity analyses | Results, Congenital Malformations in Children of TGCC Men, §2 | “In the sensitivity analyses excluding children conceived by assisted reproduction, we found negligible differences in risk estimates (all malformations OR = 1.25, 95% CI = 1.16 to 1.36, p=0.004, 4.3% vs. 3.4%; major malformations OR = 1.37, 95% CI = 1.25 to 1.51, p < 0.001, 2.9% vs. 2.1%). Excluding from the reference group the children of fathers with non-TGCC cancer did not change the risk estimates (OR = 1.28, 95% CI = 1.19 to 1.38, p=0.001, 4.4% vs. 3.5% and OR = 1.36, 95% CI = 1.24 to 1.49, p < 0.001, 2.9% vs. 2.2%).” |
| --- | --- | --- | --- | --- |
| Discussion | | | | |
| Key results | 18 | Summarise key results with reference to study objectives | Discussion, §1 | “Although children born to fathers with TGCC have a significantly increased risk of all and major CM, it is unlikely to be due to the effects of radio- or chemotherapy. There was no difference in risk estimates between children conceived after radio- or chemotherapy as compared to those conceived prior to these potentially mutagenic treatments. Although very few in numbers, the same was true even for children conceived after more extensive paternal chemotherapy regimens.” |
| Limitations | 19 | Discuss limitations of the study, taking into account sources of potential bias or imprecision. Discuss both direction and magnitude of any potential bias | Discussion, §8  Discussion, §9 | “The strength of this study is the utilization of large register data which made it possible to estimate malformation risk in children conceived prior to as well as after TGCC diagnoses. Through linking the national registries with SWENOTECA, we have got access to complete and detailed treatment data. Although the data on seminoma patients are lacking for the period 1995-2000, misclassification due to inclusion of children of those men in the control group should rather lead to diminishing the difference in malformation risk between TGCC-offspring and the non-TGCC-offspring. Despite the use of national registries, the number of children with malformations fathered by men who received radiotherapy or very intensive chemotherapy treatment was still rather low. Therefore, the results for these subgroups should be taken with some caution. “  “This study also had several limitations. Data on seminoma patients are lacking for the period 1995-2000. However, misclassification due to inclusion of children of those men in the control group should rather lead to diminishing the difference in malformation risk between TGCC-offspring and the non-TGCC-offspring. Furthermore, despite the use of national registries, the number of children with malformations fathered by men who received radiotherapy or very intensive chemotherapy treatment was still rather low. Therefore, the results for these subgroups should be taken with some caution. Another limitation is the lack of data regarding children born after insemination with cryopreserved or donor sperm. This was mitigated though the sensitivity analysis where children born after assisted reproduction were excluded, which showed the same result” |
| Interpretation | 20 | Give a cautious overall interpretation of results considering objectives, limitations, multiplicity of analyses, results from similar studies, and other relevant evidence | Discussion, §7  Discussion, §9 | “Our findings have some potential clinical implications. First of all, although we find somewhat increased risk of CM in children fathered by men with TGCC, this increase is rather modest and this reassuring information can be passed on to the patients. [...] despite the use of national registries, the number of children with malformations fathered by men who received radiotherapy or very intensive chemotherapy treatment was still rather low. Therefore, the results for these subgroups should be taken with some caution.” |
| Generalisability | 21 | Discuss the generalisability (external validity) of the study results | Discussion, §8 | “The strength of this study is the utilization of large register data which made it possible to estimate malformation risk in children conceived prior to as well as after TGCC diagnoses. Through linking the national registries with SWENOTECA, we have got access to complete and detailed treatment data.” |
| Other information | |  | | |
| Funding | 22 | Give the source of funding and the role of the funders for the present study and, if applicable, for the original study on which the present article is based | Title-page, Funding, , §1 | The study was supported by funds from ReproUnion (EU Interreg V program), Swedish Cancer Society, Swedish Childhood Cancer Society, Swedish Governmental Funding (ALF), Malmö University Hospital Cancer Fund, and Nordic Cancer Union |

*Give information separately for cases and controls in case-control studies and, if applicable, for exposed and unexposed groups in cohort and cross-sectional studies.

**Note:** An Explanation and Elaboration article discusses each checklist item and gives methodological background and published examples of transparent reporting. The STROBE checklist is best used in conjunction with this article (freely available on the Web sites of PLoS Medicine at http://www.plosmedicine.org/, Annals of Internal Medicine at http://www.annals.org/, and Epidemiology at http://www.epidem.com/). Information on the STROBE Initiative is available at www.strobe-statement.org.
