# Supplementary material for: Cancer therapy and risk of congenital malformations in children fathered by men treated for testicular germ-cell cancer: A nationwide register study
Source: PLoS Med. 2019 Jun 4;16(6):e1002816. doi: 10.1371/journal.pmed.1002816 (PMC6548355; doi:10.1371/journal.pmed.1002816)
Supplement: S1 Table — (DOCX) [file pmed.1002816.s002.docx]

| S1 Table. Frequency and risk estimates for all and major congenital malformations for children stratified according to the number of chemotherapy cycles the father has been treated with | | | | |
| --- | --- | --- | --- | --- |
|  | Father treated with chemotherapy | | | |
|  | Child conceived prior to chemotherapy | Child conceived after chemotherapy | Odds Ratio (95% Confidence Interval) | P value |
| ***Nr of chemotherapy cycles*** |  |  |  |  |
| *1-2 cycles* |  |  |  |  |
| *Nr of children* | 1061 | 502 |  |  |
| *Assisted mode of conception* | 31 | 66 |  |  |
| All congenital abnormalities, No. (%) | 47 (4.4) | 21 (4.2) | 0.88 (0.51 – 1.53) | 0.66 |
| Major congenital abnormalities, No. (%) | 31 (2.9) | 18 (3.6) | 1.25 (0.67 - 2.32) | 0.48 |
| *3-4 cycles* |  |  |  |  |
| *Nr of children* | 510 | 354 |  |  |
| *Assisted mode of conception* | 7 | 65 |  |  |
| All congenital abnormalities, No. (%) | 22 (4.3) | 14 (4.0) | 0.81 (0.39 - 1.65) | 0.55 |
| Major congenital abnormalities, No. (%) | 14 (2.7) | 8 (2.3) | 0.80 (0.32 - 2.00) | 0.64 |
| *5+ cycles* |  |  |  |  |
| *Nr of children* | 35 | 34 |  |  |
| *Assisted mode of conception* | 2 | 7 |  |  |
| All congenital abnormalities, No. (%) | 4 (11.4) | 2 (5.9) | 0.45 (0.06 - 3.44) | 0.44 |
| Major congenital abnormalities, No. (%) | 3 (8.6) | 2 (5.9) | 0.65 (0.08 - 5.28) | 0.69 |
| *Missing Data* | 34 | 4 |  |  |
| *Total No. of children* | 1639 | 894 |  |  |

*All presented risk estimates did not change substantially when excluding children conceived via assisted reproduction.*
