# Supplementary material for: Cancer therapy and risk of congenital malformations in children fathered by men treated for testicular germ-cell cancer: A nationwide register study
Source: PLoS Med. 2019 Jun 4;16(6):e1002816. doi: 10.1371/journal.pmed.1002816 (PMC6548355; doi:10.1371/journal.pmed.1002816)
Supplement: S2 Table — (DOCX) [file pmed.1002816.s003.docx]

| S2 Table. Crude and adjusted risk estimates for all and major congenital malformations | | | | |
| --- | --- | --- | --- | --- |
| **Analysis** | Crude Odds Ratio(95% CI) | P Value | Adjusted Odds Ratio (95% CI), In Paper | P Value |
| **Congenital Malformations in Children of TGCC Men as compared to non-TGCC** |  |  |  |  |
| All malformations | 1.28 (1.10 - 1.48) | 0.001 | 1.28 (1.19 - 1.38) | 0.001 |
| Major Malformations | 1.35 (1.13 - 1.62) | 0.001 | 1.36 (1.24 - 1.49) | < 0.001 |
| *Excluding children conceived by assisted reproduction* |  |  |  |  |
| All malformations | 1.25 (1.07 - 1.46) | 0.004 | 1.25 (1.16 - 1.36) | 0.004 |
| Major Malformations | 1.37 (1.14 - 1.65) | < 0.001 | 1.37 (1.25 - 1.51) | < 0.001 |
| **Children conceived after paternal TGCC diagnosis as compared to those conceived before paternal TGCC** |  |  |  |  |
| All malformations | 0.91 (0.66 - 1.24) | 0.54 | 0.88 (0.63 - 1.22) | 0.43 |
| Major Malformations | 1.01 (0.69 - 1.48) | 0.95 | 1.03 (0.69 - 1.53) | 0.88 |
| **Children conceived after paternal radiotherapy as compared to those conceived before paternal radiotherapy** |  |  |  |  |
| All malformations | 1.08 (0.32 - 3.61) | 0.90 | 1.01 (0.25 - 4.12) | 0.98 |
| Major Malformations | 1.30 (0.32 - 5.28) | 0.71 | 1.37 (0.27 - 7.05) | 0.70 |
| **Children conceived after paternal chemotherapy as compared to those conceived before paternal chemotherapy** |  |  |  |  |
| All malformations | 0.90 (0.60 - 1.35) | 0.61 | 0.82 (0.54 - 1.25) | 0.37 |
| Major Malformations | 1.03 (0.64 - 1.64) | 0.91 | 1.01 (0.62 - 1.65) | 0.97 |
