# Supplementary material for: Cancer therapy and risk of congenital malformations in children fathered by men treated for testicular germ-cell cancer: A nationwide register study
Source: PLoS Med. 2019 Jun 4;16(6):e1002816. doi: 10.1371/journal.pmed.1002816 (PMC6548355; doi:10.1371/journal.pmed.1002816)
Supplement: S3 Table — (DOCX) [file pmed.1002816.s004.docx]

| S3 Table. Pooled risk estimates for all variables in the model comparing children conceived after paternal radiotherapy to children conceived before paternal radiotherapy | | | | |
| --- | --- | --- | --- | --- |
|  |  | Confidence interval | |  |
| **Characteristic** | Odds ratio | Lower | Upper | P value |
| ***All malformations*** |  |  |  |  |
| Paternal age at offspring birth, years | 0.974 | 0.823 | 1.153 | 0.762 |
| Maternal age at childbirth, years | 1.068 | 0.892 | 1.279 | 0.475 |
| Maternal smoking, nonsmoker | ref |  |  |  |
| Maternal smoking, 1-9 cigarettes per day | 0.000 | 0.000 | . | 0.999 |
| Maternal smoking, ≥10 cigarettes per day | 5.442 | 0.293 | 100.940 | 0.255 |
| Maternal BMI, <20 kg/m^2^ | ref |  |  |  |
| Maternal BMI, ≥20 to <25 kg/m^2^ | 0.743 | 0.079 | 6.970 | 0.794 |
| Maternal BMI, ≥25 to <30 kg/m^2^ | 2.180 | 0.212 | 22.449 | 0.511 |
| Maternal BMI,≥30 to <35 kg/m^2^ | 0.000 | 0.000 | . | 0.998 |
| Maternal BMI, ≥35 kg/m^2^ | 2.541 | 0.091 | 71.213 | 0.583 |
| Child conceived before radiotherapy | ref |  |  |  |
| Child conceived after radiotherapy | 1.014 | 0.250 | 4.118 | 0.984 |
| ***Major Malformations*** |  |  |  |  |
| Paternal age at offspring birth, years | 0.937 | 0.769 | 1.141 | 0.516 |
| Maternal age at childbirth, years | 1.074 | 0.872 | 1.323 | 0.501 |
| Maternal smoking, nonsmoker | ref |  |  |  |
| Maternal smoking, 1-9 cigarettes per day | 0.000 | 0.000 | 0.000 | 1.000 |
| Maternal smoking, ≥10 cigarettes per day | 6.511 | 0.321 | 131.960 | 0.222 |
| Maternal BMI, <20 kg/m^2^ | ref |  |  |  |
| Maternal BMI, ≥20 to <25 kg/m^2^ | 0.422 | 0.037 | 4.837 | 0.488 |
| Maternal BMI, ≥25 to <30 kg/m^2^ | 2.271 | 0.234 | 22.046 | 0.479 |
| Maternal BMI, ≥30 to <35 kg/m^2^ | 0.000 | 0.000 | 0.000 | 1.000 |
| Maternal BMI, ≥35 kg/m^2^ | 2.898 | 0.097 | 86.405 | 0.539 |
| Child conceived before radiotherapy | ref |  |  |  |
| Child conceived after radiotherapy | 1.373 | 0.267 | 7.046 | 0.704 |

*Abbreviations: BMI, body mass index.*
