# Supplementary material for: Cancer therapy and risk of congenital malformations in children fathered by men treated for testicular germ-cell cancer: A nationwide register study
Source: PLoS Med. 2019 Jun 4;16(6):e1002816. doi: 10.1371/journal.pmed.1002816 (PMC6548355; doi:10.1371/journal.pmed.1002816)
Supplement: S4 Table — (DOCX) [file pmed.1002816.s005.docx]

| S4 Table. Pooled risk estimates for all variables in the model comparing children conceived after paternal chemotherapy to children conceived before paternal chemotherapy | | | | |
| --- | --- | --- | --- | --- |
|  |  | Confidence interval | |  |
| **Characteristic** | Odds ratio | Lower | Upper | P value |
| ***All malformations*** |  |  |  |  |
| Paternal age at offspring birth, years | 1.012 | 0.957 | 1.069 | 0.683 |
| Maternal age at childbirth, years | 1.010 | 0.956 | 1.068 | 0.715 |
| Maternal smoking, nonsmoker | ref |  |  |  |
| Maternal smoking, 1-9 cigarettes per day | 0.485 | 0.150 | 1.566 | 0.226 |
| Maternal smoking, ≥10 cigarettes per day | 0.000 | 0.000 | 0.000 | 1.000 |
| Maternal BMI, <20 kg/m^2^ | ref |  |  |  |
| Maternal BMI, ≥20 to <25 kg/m^2^ | 1.251 | 0.617 | 2.537 | 0.534 |
| Maternal BMI, ≥25 to <30 kg/m^2^ | 1.432 | 0.664 | 3.088 | 0.359 |
| Maternal BMI, ≥30 to <35 kg/m^2^ | 1.167 | 0.429 | 3.176 | 0.762 |
| Maternal BMI, ≥35 kg/m^2^ | 0.000 | 0.000 | . | 0.997 |
| Child conceived before chemotherapy | ref |  |  |  |
| Child conceived after chemotherapy | 0.824 | 0.543 | 1.252 | 0.366 |
| ***Major Malformations*** |  |  |  |  |
| Paternal age at offspring birth, years | 0.994 | 0.930 | 1.063 | 0.866 |
| Maternal age at childbirth, years | 1.008 | 0.944 | 1.077 | 0.811 |
| Maternal smoking, nonsmoker | ref |  |  |  |
| Maternal smoking, 1-9 cigarettes per day | 0.704 | 0.215 | 2.306 | 0.562 |
| Maternal smoking, ≥10 cigarettes per day | 0.000 | 0.000 | 0.000 | 1.000 |
| Maternal BMI, <20 kg/m^2^ | ref |  |  |  |
| Maternal BMI, ≥20 to <25 kg/m^2^ | 1.054 | 0.470 | 2.365 | 0.899 |
| Maternal BMI, ≥25 to <30 kg/m^2^ | 1.354 | 0.589 | 3.113 | 0.476 |
| Maternal BMI, ≥30 to <35 kg/m^2^ | 1.028 | 0.330 | 3.204 | 0.962 |
| Maternal BMI, ≥35 kg/m^2^ | 0.000 | 0.000 | 0.000 | 1.000 |
| Child conceived before chemotherapy | ref |  |  |  |
| Child conceived after chemotherapy | 1.009 | 0.618 | 1.647 | 0.971 |

*Abbreviations: BMI, body mass index.*
