# Supplementary material for: Cancer therapy and risk of congenital malformations in children fathered by men treated for testicular germ-cell cancer: A nationwide register study
Source: PLoS Med. 2019 Jun 4;16(6):e1002816. doi: 10.1371/journal.pmed.1002816 (PMC6548355; doi:10.1371/journal.pmed.1002816)
Supplement: S5 Table — (DOCX) [file pmed.1002816.s006.docx]

| S5 Table. Pooled risk estimates for all variables in the model comparing children conceived after paternal TGCC diagnosis as compared to those conceived before paternal TGCC | | | | |
| --- | --- | --- | --- | --- |
|  |  | Confidence interval | |  |
| **Characteristic** | Odds ratio | Lower | Upper | P value |
| ***All malformations*** |  |  |  |  |
| Paternal age at offspring birth, years | 0.985 | 0.943 | 1.028 | 0.483 |
| Maternal age at childbirth, years | 1.028 | 0.985 | 1.074 | 0.203 |
| Maternal smoking, nonsmoker | ref |  |  |  |
| Maternal smoking, 1-9 cigarettes per day | 0.657 | 0.305 | 1.417 | 0.283 |
| Maternal smoking, ≥10 cigarettes per day | 0.462 | 0.113 | 1.879 | 0.279 |
| Maternal BMI, <20 kg/m^2^ | ref |  |  |  |
| Maternal BMI, ≥20 to <25 kg/m^2^ | 1.075 | 0.648 | 1.782 | 0.780 |
| Maternal BMI, ≥25 to <30 kg/m^2^ | 1.179 | 0.657 | 2.116 | 0.580 |
| Maternal BMI, ≥30 to <35 kg/m^2^ | 1.038 | 0.474 | 2.272 | 0.925 |
| Maternal BMI, ≥35 kg/m^2^ | 0.673 | 0.199 | 2.277 | 0.524 |
| Child conceived before TGCC | ref |  |  |  |
| Child conceived after TGCC | 0.876 | 0.63 | 1.217 | 0.429 |
| ***Major Malformations*** |  |  |  |  |
| Paternal age at offspring birth, years | 0.979 | 0.929 | 1.032 | 0.423 |
| Maternal age at childbirth, years | 1.017 | 0.965 | 1.072 | 0.518 |
| Maternal smoking, nonsmoker | ref |  |  |  |
| Maternal smoking, 1-9 cigarettes per day | 0.940 | 0.420 | 2.104 | 0.880 |
| Maternal smoking, ≥10 cigarettes per day | 0.389 | 0.054 | 2.798 | 0.346 |
| Maternal BMI, <20 kg/m^2^ | ref |  |  |  |
| Maternal BMI, ≥20 to <25 kg/m^2^ | 0.906 | 0.494 | 1.660 | 0.749 |
| Maternal BMI, ≥25 to <30 kg/m^2^ | 1.199 | 0.617 | 2.332 | 0.591 |
| Maternal BMI, ≥30 to <35 kg/m^2^ | 1.165 | 0.489 | 2.776 | 0.730 |
| Maternal BMI, ≥35 kg/m^2^ | 0.888 | 0.248 | 3.187 | 0.856 |
| Child conceived before TGCC | ref |  |  |  |
| Child conceived after TGCC | 1.031 | 0.694 | 1.532 | 0.878 |

*Abbreviations: BMI, body mass index; TGCC, testicular germ cell cancer.*
