# Supplementary material for: Cancer therapy and risk of congenital malformations in children fathered by men treated for testicular germ-cell cancer: A nationwide register study
Source: PLoS Med. 2019 Jun 4;16(6):e1002816. doi: 10.1371/journal.pmed.1002816 (PMC6548355; doi:10.1371/journal.pmed.1002816)
Supplement: S6 Table — (DOCX) [file pmed.1002816.s007.docx]

| S6 Table. Pooled risk estimates for all variables in the model comparing children conceived to fathers with TGCC as compared to those children born to fathers without TGCC | | | | |
| --- | --- | --- | --- | --- |
|  |  | Confidence interval | |  |
| **Characteristic** | Odds ratio | Lower | Upper | P value |
| ***All malformations*** |  |  |  |  |
| Paternal age at offspring birth, years | 0.998 | 0.997 | 1.000 | 0.064 |
| Maternal age at childbirth, years | 1.006 | 1.004 | 1.008 | <0.001 |
| Maternal smoking, nonsmoker | ref |  |  |  |
| Maternal smoking, 1-9 cigarettes per day | 1.013 | 0.983 | 1.044 | 0.407 |
| Maternal smoking. ≥10 cigarettes per day | 1.022 | 0.975 | 1.071 | 0.362 |
| Maternal BMI, <20 kg/m^2^ | ref |  |  |  |
| Maternal BMI, ≥20 to <25 kg/m^2^ | 1.008 | 0.983 | 1.035 | 0.535 |
| Maternal BMI, ≥25 to <30 kg/m^2^ | 1.039 | 1.009 | 1.069 | 0.010 |
| Maternal BMI, ≥30 to <35 kg/m^2^ | 1.071 | 1.033 | 1.111 | <0.001 |
| Maternal BMI, ≥35 kg/m^2^ | 1.127 | 1.074 | 1.183 | <0.001 |
| Child conceived to fathers without TGCC | ref |  |  |  |
| Child conceived to fathers with TGCC | 1.279 | 1.186 | 1.379 | 0.001 |
| ***Major Malformations*** |  |  |  |  |
| Paternal age at offspring birth, years | 1.000 | 0.998 | 1.002 | 0.681 |
| Maternal age at childbirth, years | 1.008 | 1.005 | 1.010 | <0.001 |
| Maternal smoking, nonsmoker | ref |  |  |  |
| Maternal smoking, 1-9 cigarettes per day | 1.028 | 0.989 | 1.069 | 0.161 |
| Maternal smoking, ≥10 cigarettes per day | 1.044 | 0.986 | 1.105 | 0.137 |
| Maternal BMI, <20 kg/m^2^ | ref |  |  |  |
| Maternal BMI, ≥20 to <25 kg/m^2^ | 1.021 | 0.988 | 1.054 | 0.217 |
| Maternal BMI, ≥25 to <30 kg/m^2^ | 1.084 | 1.045 | 1.123 | <0.001 |
| Maternal BMI, ≥30 to <35 kg/m^2^ | 1.133 | 1.084 | 1.185 | <0.001 |
| Maternal BMI, ≥35 kg/m^2^ | 1.258 | 1.186 | 1.335 | <0.001 |
| Child conceived to fathers without TGCC | ref |  |  |  |
| Child conceived to fathers with TGCC | 1.356 | 1.237 | 1.486 | <0.001 |

*Abbreviations: BMI, body mass index; TGCC, testicular germ cell cancer.*
