# Supplementary material for: Cancer therapy and risk of congenital malformations in children fathered by men treated for testicular germ-cell cancer: A nationwide register study
Source: PLoS Med. 2019 Jun 4;16(6):e1002816. doi: 10.1371/journal.pmed.1002816 (PMC6548355; doi:10.1371/journal.pmed.1002816)
Supplement: S7 Table — (DOCX) [file pmed.1002816.s008.docx]

| S7 Table. Pooled risk estimates for all variables in the model comparing children conceived to fathers with TGCC as compared to those children born to fathers without TGCC, whilst excluding all children conceived through assisted reproductive techniques | | | | |
| --- | --- | --- | --- | --- |
|  |  | Confidence interval | |  |
| **Characteristic** | Odds ratio | Lower | Upper | P value |
| ***All malformations*** |  |  |  |  |
| Paternal age at offspring birth, years | 0.998 | 0.997 | 1.000 | 0.049 |
| Maternal age at childbirth, years | 1.006 | 1.004 | 1.008 | <0.001 |
| Maternal smoking, nonsmoker | ref |  |  |  |
| Maternal smoking, 1-9 cigarettes per day | 1.014 | 0.983 | 1.045 | 0.389 |
| Maternal smoking, ≥10 cigarettes per day | 1.024 | 0.977 | 1.073 | 0.329 |
| Maternal BMI, <20 kg/m^2^ | ref |  |  |  |
| Maternal BMI, ≥20 to <25 kg/m^2^ | 1.007 | 0.981 | 1.034 | 0.589 |
| Maternal BMI, ≥25 to <30 kg/m^2^ | 1.038 | 1.008 | 1.069 | 0.012 |
| Maternal BMI, ≥30 to <35 kg/m^2^ | 1.069 | 1.030 | 1.109 | <0.001 |
| Maternal BMI, ≥35 kg/m^2^ | 1.127 | 1.073 | 1.183 | <0.001 |
| Child conceived to fathers without TGCC | ref |  |  |  |
| Child conceived to fathers with TGCC | 1.253 | 1.158 | 1.356 | 0.004 |
| ***Major Malformations*** |  |  |  |  |
| Paternal age at offspring birth, years | 0.999 | 0.997 | 1.002 | 0.627 |
| Maternal age at childbirth, years | 1.007 | 1.005 | 1.010 | <0.001 |
| Maternal smoking, nonsmoker | ref |  |  |  |
| Maternal smoking, 1-9 cigarettes per day | 1.029 | 0.989 | 1.070 | 0.154 |
| Maternal smoking, ≥10 cigarettes per day | 1.043 | 0.985 | 1.105 | 0.148 |
| Maternal BMI, <20 kg/m^2^ | ref |  |  |  |
| Maternal BMI, ≥20 to <25 kg/m^2^ | 1.018 | 0.985 | 1.052 | 0.284 |
| Maternal BMI, ≥25 to <30 kg/m^2^ | 1.081 | 1.043 | 1.121 | <0.001 |
| Maternal BMI, ≥30 to <35 kg/m^2^ | 1.130 | 1.080 | 1.182 | <0.001 |
| Maternal BMI, ≥35 kg/m^2^ | 1.258 | 1.185 | 1.336 | <0.001 |
| Child conceived to fathers without TGCC | ref |  |  |  |
| Child conceived to fathers with TGCC | 1.374 | 1.250 | 1.511 | 0.001 |

*Abbreviations: BMI, body mass index; TGCC, testicular germ cell cancer.*
