# Supplementary material for: Cancer therapy and risk of congenital malformations in children fathered by men treated for testicular germ-cell cancer: A nationwide register study
Source: PLoS Med. 2019 Jun 4;16(6):e1002816. doi: 10.1371/journal.pmed.1002816 (PMC6548355; doi:10.1371/journal.pmed.1002816)
Supplement: S9 Table — (DOCX) [file pmed.1002816.s010.docx]

| S9 Table. Missing and imputed values in the original and the 5 imputed datasets | | | | | | |
| --- | --- | --- | --- | --- | --- | --- |
|  |  | Imputation | | | | |
| **Variable** | Original data | 1 | 2 | 3 | 4 | 5 |
| ***Maternal age at childbirth, years*** |  |  |  |  |  |  |
| Valid values | 2027931 | 2027997 | 2027997 | 2027997 | 2027997 | 2027997 |
| Missing data | 66 | 0 | 0 | 0 | 0 | 0 |
| Mean | 29.886 | 29.886 | 29.886 | 29.886 | 29.886 | 29.886 |
| SD | 5.133 | 5.133 | 5.133 | 5.133 | 5.133 | 5.133 |
| P value | - | 0.998 | 0.998 | 1.000 | 0.995 | 0.994 |
| ***Maternal weight, kg*** |  |  |  |  |  |  |
| Valid values | 1827539 | 2027997 | 2027997 | 2027997 | 2027997 | 2027997 |
| Missing data | 200458 | 0 | 0 | 0 | 0 | 0 |
| Mean | 67.623 | 67.633 | 67.629 | 67.641 | 67.638 | 67.628 |
| SD | 12.977 | 12.980 | 12.976 | 12.973 | 12.976 | 12.977 |
| P value | - | 0.463 | 0.634 | 0.174 | 0.264 | 0.687 |
| ***Maternal height, cm*** |  |  |  |  |  |  |
| Valid values | 1901181 | 2027997 | 2027997 | 2027997 | 2027997 | 2027997 |
| Missing data | 126816 | 0 | 0 | 0 | 0 | 0 |
| Mean | 166.311 | 166.305 | 166.304 | 166.303 | 166.304 | 166.305 |
| SD | 6.367 | 6.367 | 6.368 | 6.371 | 6.368 | 6.366 |
| P value | - | 0.329 | 0.238 | 0.185 | 0.237 | 0.358 |
| ***Maternal smoking*** |  |  |  |  |  |  |
| Valid values | 1926676 | 2027997 | 2027997 | 2027997 | 2027997 | 2027997 |
| Missing data | 101321 | 0 | 0 | 0 | 0 | 0 |
| Non-smoking mothers early in pregnancy | 1738900 | 1830818 | 1830628 | 1830867 | 1830677 | 1830783 |
| Mothers smoking 1-9 cigarettes per day | 133336 | 139977 | 140164 | 139955 | 140105 | 140045 |
| Mothers smoking more than 10 cigarettes per day | 54440 | 57202 | 57205 | 57175 | 57215 | 57169 |
| P value | - | 0.732 | 0.896 | 0.688 | 0.861 | 0.770 |

*Continuous variables (all but maternal smoking) were tested by independent student t-test by comparing each imputed dataset to the original data. Maternal smoking was tested by Pearson Chi-Square statistic.*
