# Supplementary material for: Cancer therapy and risk of congenital malformations in children fathered by men treated for testicular germ-cell cancer: A nationwide register study
Source: PLoS Med. 2019 Jun 4;16(6):e1002816. doi: 10.1371/journal.pmed.1002816 (PMC6548355; doi:10.1371/journal.pmed.1002816)
Supplement: S1 Text — (DOCX) [file pmed.1002816.s011.docx]

# Impact of cancer therapy on risk of congenital malformations in children fathered by men treated for testicular germ cell cancer: a nationwide register study

# Analysis Plan

Yahia Al-Jebari
2019-03-21

**Background (In Paper)**

Children fathered by men treated for cancer might be at higher risk for congenital malformations (CM) due to mutagenic effects of irradiation and cytotoxic drugs. Adverse effects of oncological treatments on germ cells has been described in animal [1,2] and human studies [3–5]. Furthermore, a Danish-Swedish population-based study has shown that children conceived after a father’s cancer diagnosis have a slight increase in prevalence of severe CM [6]. A possible pathway for the increased malformations risk might be detrimental genetic alterations of germline DNA by oncological treatment, resulting in more frequent CM in children conceived after paternal oncological treatment.

There are indications that the excess risk of congenital malformations for children born to fathers with cancer might be due to the malignancy *per se* rather than to the anti-cancer treatment. A Swedish population-based register study including 2.1 million children investigated the congenital malformation risk for children conceived prior to and after paternal cancer diagnosis and found those two groups to have an increased risk of malformations of about the same magnitude [7]. This study, when stratifying on cancer type, specifically showed that testicular cancer is one of the malignancies associated with increased CM risk in children conceived prior to paternal cancer diagnosis.

Testicular cancer is the most common cancer in young men and approximately 97% of testicular cancers are derived from germ cells, so called testicular germ cell cancer (TGCC). TGCC has a 15 year survival of about 95% [8], and most patients will father children before or after the diagnosis. Among those TGCC patients who are childless at the time of cancer diagnosis, 77% express wish of future fatherhood, despite some having anxieties about potential detrimental health effects of cancer therapy on the health of their offspring [9].

Previous studies have lacked treatment data making estimation of the possible additional effects of specific cancer therapies impossible. Opportunely, The Swedish Norwegian Testicular Cancer Group (SWENOTECA) registry includes treatment data on non-seminoma patients treated since 1995 and seminoma patients since 2000 for all Swedish and Norwegian TGCC patients. The main aim of this study was, by linking Swedish national registries to SWENOTECA, to investigate whether anti-neoplastic therapy implies any additional malformation risk in children fathered by men treated for TGCC. The secondary aim was to investigate whether TCGG *per se* is associated with risk of CM.

**Study Aims**

- To investigate whether paternal anti-neoplastic therapy is associated with malformation risk in children fathered by men treated for TGCC
  - Effects of paternal chemotherapy exposure (yes/no)
    - Effects of dose of chemotherapy (cycles of chemotherapy)
  - Effects of paternal radiotherapy exposure (yes/no)
  - Effects of paternal any (combined) treatment
- To investigate whether paternal TCGG is associated with risk of CM.

**Study Design and Data Sources**

This study was based on register data supplied by the Swedish Board of Health and Welfare in two sections. The first section contained data from the Medical Birth Register, Swedish Total Population Register, the Swedish Multigenerational Register, and the Swedish National Quality Register for Assisted Reproduction. This section defined the cohort and contained data on all children registered in the Medical Birth Register and born alive in Sweden during 1994–2014 (n = 2 108 569) and their parents. This data is described in depth elsewhere [7].

The second section contained data also supplied by the Swedish Board of Health and Welfare. This section contained data from the SWENOTECA registry such as paternal TGCC diagnoses and treatment information. SWENOTECA holds information on clinical stage, treatment, and follow-up for up to 10 years after diagnosis, for non-seminoma patients since 1995 and seminoma patients since 2000.

The Swedish Board of Health and Welfare merged these two datasets on the Swedish personal Identity Number and supplied us with anonymized excerpts. Of the 1 167 665 fathers in the cohort, 2380 had been diagnosed with TGCC. And of the 2 027 997 children, 4207 children had fathers with TGCC.

**Missing data**

As noticed in our pervious paper on the same cohort, around 13% of cases are lost when using full-case analysis due to missing data, primarily due to missing maternal weight and height [7]. To avoid loss of cases and therefore power, multiple imputation is used to impute these missing data.

**Outcomes**

- All malformations
  - The definition used for all congenital abnormalities was: ICD-9-SE 740-759 and ICD-10-SE Q00-Q99.
- Major malformations
  - Classified following the coding guide of European Surveillance of Congenital Anomalies as has been described previously [7]

**Statistical analysis**

Descriptive data was tabulated for the children according to TGCC-status and when they were conceived in relation to paternal TGCC diagnosis. For the children to fathers with TGCC, descriptive data was also given stratified along paternal treatment modality. Further descriptive data, such as the distribution of fathers according to TGCC subtype (seminoma/nonseminoma) and treatment modality was included. Odds ratios for congenital malformation was estimated by binary logistic regression.

Statistical analyses were preformed using SPSS version 25 (IBM Corp, Armonk, NY). All statistical analyses were two-sided; P values of less than .05 were considered statistically significant.

As there is some evidence [7] that TGCC might be associated with offspring malformations, the analyses were designed to adjust for the presence of TGCC:

- Effects of paternal chemotherapy exposure
  - Compare children conceived to fathers after exposure to chemotherapy to those children conceived before exposure to chemotherapy
- Effects of dose of chemotherapy
  - Compare children conceived to fathers after a certain dose of chemotherapy to those children conceived before the same dose of chemotherapy
- Effects of paternal radiotherapy exposure
  - Compare children conceived to fathers after exposure to radiotherapy to those children conceived before exposure to radiotherapy
- Effects of paternal any (combined) treatment
  - Compare children conceived to fathers after paternal TGCC diagnosis to those children conceived before paternal TGCC diagnosis
- To investigate whether paternal TCGG is associated with risk of CM
  - Compare children conceived to fathers with TGCC diagnosis to those children conceived to fathers without TGCC diagnosis

These analyses were planned July 2017. The SWENOTECA data was received in Oct 2017 with analyses commencing thereafter.

**References**

1. Mughal SK, Myazin AE, Zhavoronkov LP, Rubanovich A V., Dubrova YE. The dose and dose-rate effects of paternal irradiation on transgenerational instability in mice: A radiotherapy connection. Woloschak GE, editor. PLoS One. Public Library of Science; 2012;7: e41300. doi:10.1371/journal.pone.0041300

2. Witt KL, Bishop JB. Mutagenicity of anticancer drugs in mammalian germ cells. Mutat Res - Fundam Mol Mech Mutagen. 1996;355: 209–234. doi:10.1016/0027-5107(96)00029-2

3. Thomson AB, Campbell AJ, Irvine DC, Anderson RA, Kelnar CJ, Wallace WH. Semen quality and spermatozoal DNA integrity in survivors of childhood cancer: a case-control study. Lancet. 2002;360: 361–367. doi:10.1016/S0140-6736(02)09606-X

4. Stahl O, Eberhard J, Jepson K, Spano M, Cwikiel M, Cavallin-Stahl E, et al. Sperm DNA integrity in testicular cancer patients. Hum Reprod. Oxford University Press; 2006;21: 3199–3205. doi:10.1093/humrep/del292

5. Spermon JR, Ramos L, Wetzels AMM, Sweep CGJ, Braat DDM, Kiemeney LALM, et al. Sperm integrity pre- and post-chemotherapy in men with testicular germ cell cancer. Hum Reprod. 2006;21: 1781–1786. doi:10.1093/humrep/del084

6. Ståhl O, Boyd HA, Giwercman A, Lindholm M, Jensen A, Krüger Kjær S, et al. Risk of birth abnormalities in the offspring of men with a history of cancer: A cohort study using danish and swedish national registries. J Natl Cancer Inst. Oxford University Press; 2011;103: 398–406. doi:10.1093/jnci/djq550

7. Al-Jebari Y, Rylander L, Ståhl O, Giwercman A. Risk of Congenital Malformations in Children Born Before Paternal Cancer. JNCI Cancer Spectr. Oxford University Press; 2018;2. doi:10.1093/jncics/pky027

8. Miller KD, Siegel RL, Lin CC, Mariotto AB, Kramer JL, Rowland JH, et al. Cancer treatment and survivorship statistics, 2016. CA Cancer J Clin. American Cancer Society; 2016;66: 271–289. doi:10.3322/caac.21349

9. Schover LR, Brey K, Lichtin A, Lipshultz LI, Jeha S. Knowledge and experience regarding cancer, infertility, and sperm banking in younger male survivors. J Clin Oncol. American Society of Clinical Oncology; 2002;20: 1880–9. doi:10.1200/JCO.2002.07.175
