# Supplementary material for: Cancer therapy and risk of congenital malformations in children fathered by men treated for testicular germ-cell cancer: A nationwide register study
Source: PLoS Med. 2019 Jun 4;16(6):e1002816. doi: 10.1371/journal.pmed.1002816 (PMC6548355; doi:10.1371/journal.pmed.1002816)
Supplement: S2 Text — (DOCX) [file pmed.1002816.s012.docx]

2015-09-29 version 1

**Hälsoaspekter hos barn vars fäder behandlats för cancer. En uppdatering.**

**Paternal cancer and health of the offspring – an update register study.**

**Study coordinator:**

Aleksander Giwercman, Lund University: [aleksander.giwercman@med.lu.se](mailto:aleksander.giwercman@med.lu.se)

**Other investigators**

Matilda Martinsson: [matilda.martinsson@hotmail.com](mailto:matilda.martinsson@hotmail.com)

Lars Rylander: [lars.rylander@med.lu.se](mailto:lars.rylander@med.lu.se)

**Background**

In a study based on a database of almost 1.8 million singleton children born in Denmark and Sweden during the period 1994-2005, we found that 8,670 of the children had fathers who previously had been diagnosed with cancer. These data has been generated by linking the information from several health registries. The risk for major congenital malformations was significantly increased (3.7% vs. 3.2%; Relative Risk = 1.17, 95% CI 1.05-1.31, p=0.004) in offspring of male cancer survivors as compared to the background population (1).

In this registry-based study, no information on cancer treatment was available. However, whereas a paternal history of hematological malignancy, generally treated with chemotherapy, was associated with an increased risk for congenital abnormalities, although not statistically significant, no increased risk was seen among children with a paternal history of testicular seminoma of whom the majority were treated with radiotherapy (stage I disease). In contrast, a paternal history of skin cancer, for which the standard treatment is surgical excision, was accompanied by almost 40% increased risk of congenital abnormalities. These findings suggest that the increased risk of congenital abnormalities may be related more to the paternal disease per se than to its treatment.

Biologically, genomic instability (GI) might be the possible link between both the paternal cancer and the risk for malformations among the offspring since increased GI leads to the initiation of cancer and could, via the same abnormalities in the DNA of the paternal spermatozoa, be transmitted to the child. In support of this hypothesis is the finding that paternal grandfathers and grandmothers with non-familial achondroplasia (common form of dwarfism) had significantly more cancers than maternal grandfathers and grandmothers (2). Notably, all sporadic achondroplasia cases had inherited the mutation from their father (3) with a strong link to increased paternal age (4). Also parents of children born with cleft lip/palate have an increased cancer risk (5). Finally, children with cancer have an overall increased risk of minor malformations, besides the classical cancer-predisposing syndromes (6).

Many years ago, Loeb et al proposed and developed the hypothesis that cancer cells exhibit a mutator phenotype (7). The basic premise is that normal mutation rates are insufficient to account for the multiple mutations observed in cancer cells and therefore mutations in critical genes, which have the ability to increase mutation rates, are essential to account for the large number of aberrations observed in human tumours. The mutator phenotype arising from those genes can have diverse manifestations, such as point mutations, microsatellite instability and loss of heterozygosity. The mutator hypothesis postulates that an initial mutation generates further mutations, including those in additional genetic stability genes, resulting in a cascade of mutations throughout the genome (8). A low-grade constitutional mutator phenotype that is present in persons predisposed to cancer, could also involve germ cells and manifest itself as mutations leading to malformations.

Another possible mechanism is an increase in the mutation rate during meiosis leading to genetic aberrations in the spermatozoa, which subsequently are transmitted to the offspring (9).

However, GI has not only been linked to cancer and congenital malformations, but also to pre-mature aging and to an increased risk of diseases associated with aging as e.g. diabetes and atherosclerosis (10). Thus, one can speculate that an increased level of GI can lead to a diversity of morbidities which can manifest already in childhood or early adulthood.

Both irradiation and chemotherapy might induce an increased level of GI in germ cells (11) and thereby contribute to an elevated risk of congenital malformations as well as morbidity in offspring of men treated for testicular cancer.

If GI is the biological link between paternal cancer and congenital abnormalities in the offspring, the increased risk of malformations should be observed in the children born before the father has got his cancer diagnose. Furthermore, GI might not only lead to increased risk of congenital malformations but also to increased morbidity in the childhood. Furthermore, it might also imply reduced male fertility.

These considerations lead to following scientific questions:

**Scientific questions:**

1. Offspring of men treated for cancer:
   1. Are there increased risks of congenital malformations and/or other perinatal abnormalities in the children born before the father was diagnosed with cancer ?
   2. Is there a generally increased risk of morbidities among the offspring (i.e. hospitalisation, specific diseases like diabetes, asthma/allergy, cardiovascular disease and different autoimmune diseases) – conceived before or after cancer diagnosis/treatment?
2. Is the risk of cancer increased in fathers of children conceived by assisted reproduction?

The study will be based on all registered (Medical Birth Registry) singleton births in Sweden from 1994 onwards. By linking this database to the available national registries we will try to answer the questions defined above.

.

***Data sources***

*Medical Birth Registry:* Covers nearly all (>98%) children born in Sweden. Between 1993 and 2005, IVF and ICSI treatments were given at 18 public or private clinics, with data on all treatments leading to delivery of a baby reported to the Swedish National Board of Health, and thereby to the registry.

*Total Population Registry:* The civil registration of the inhabitants of Sweden, assigning all a unique personal identification number.

*Multigenerational Registry:* Contains information on first-degree relatives of all Swedish citizens born after 1931 and still alive in 1961, or born in 1961 or later, with close to 100% coverage.

*Cancer Registry:* Mandatory reporting of cancers in Sweden since 1958, with agreement between clinical and cytological or histological diagnoses close to 100% coverage. Information includes site of tumour (ICD-7 codes), histological type (ICD-O/2 codes for 1994-2004 and ICD-O/3 codes for 2005), and basis of and date of diagnosis.

Congenital malformations: Information retrieved from the Medical Birth Registry, and supplemented with data from the *Swedish Registry of Congenital Malformations*, and the Hospital Discharge Registry.

*National patient registry (NPR):* From 1987 NPR includes all in-patient care in Sweden. NPR includes 50 million discharges for the period 1964 to 2006. The registry contains, from 2001, also outpatient visits including day surgery and psychiatric from both private and public caregivers. Primary care is not yet covered in the NPR.

*Pharmaceutical directory registry* (Läkemedelsregistret): Contains information regarding all pharmaceuticals prescribed in Sweden by physicians since 1999. The prescription is linked to the personal number of the subject to whom the medicine was prescribed.

*IVF registry:* Includes data on all IVF treatments in Sweden performed since 2007.

***Potential confounders***

Potential confounders will be selected and applied based on the literature, and their availability in the relevant population-based health registries. Potential confounders includes calendar time, age at first male infertility diagnosis, type of assisted reproductive technology (IVF and/or ICSI), if any, number of children, place of residence, maternal smoking and socioeconomic status (level of education and disposable income).

***Statistical analyses***

For outcomes not ascertained at birth, the cohort members will be followed from birth until the first of the following events: 1) event of interest; 2) death; 3) emigration; 4) designated “missing person” in the Civil Registration System; or 5) end of follow-up (December 31, 2015).

Two different kinds of analytic approaches will be used:

For outcomes ascertained at birth (success rate, risk of miscarriage, congenital malformations and other perinatal abnormalities) logistic regression models will be applied.

For cancer, number of hospitalizations and specific somatic diseases among the children, a Cox regression model will be used (conditions with follow-up time).

All analyses will be performed using SAS and R statistical software.

***Power analysis***

In our previous project including children born during the period 1994-2004 the statistical power was sufficient to show a 17% increased relative risk of congenital malformations. By updataing the database we will increase the number of children included in the study, despite the fact that only Swedish children will be included.

***Ethical considerations***

Ethical committee in Lund will be asked for permission to perform the study. The patients will be informed about the study by announcement in the newspapers (opt out).

In case of any link between the cancer disease and any of the outcomes to be included in this study is shown, this information will be of value in counselling the patients, i.e. as considers use of fresh or cryopreserved spermatozoa, as well as in development of preventive measures in relation to the disease risk in their children. Our findings may also become a “model” for future management of the offspring of cancer survivors.

Use of cancer registries gives us the possibility to answer some important clinically and biologically relevant questions without directly getting in contact with the patients and their children. Thereby creating an unnecessary worry among those subjects can be avoided.

An application to the national data protection agencies will be submitted.

***Cancer relevance***

An increasing number of young cancer patients are being cured. For that reason the aspects of their reproductive function, which may be impaired by the cancer disease *per se* as well as by its treatment is of outermost importance.

Whereas we now have a large amount of data concerning the impact of cancer and its treatment on fertility of the patients, very little is known about the implications for the health of the offspring.

Our findings will have important implications for counselling of young male cancer survivors.

If we find a link between cancer *per se* and adverse health outcome among the children, this will provide us important information about the biology of the cancer disease.

**References**

1. Stahl O, Boyd HA, Giwercman A, Lindholm M, Jensen A, Kjaer SK, et al. Risk of birth abnormalities in the offspring of men with a history of cancer: a cohort study using Danish and Swedish national registries. Journal of the National Cancer Institute. 2011;103(5):398-406.

2. Stoll C, Feingold J. Do parents and grandparents of patients with achondroplasia have a higher cancer risk? American journal of medical genetics Part A. 2004;130A(2):165-8.

3. Wilkin DJ, Szabo JK, Cameron R, Henderson S, Bellus GA, Mack ML, et al. Mutations in fibroblast growth-factor receptor 3 in sporadic cases of achondroplasia occur exclusively on the paternally derived chromosome. American journal of human genetics. 1998;63(3):711-6.

4. Risch N, Reich EW, Wishnick MM, McCarthy JG. Spontaneous mutation and parental age in humans. American journal of human genetics. 1987;41(2):218-48.

5. Zhu JL, Basso O, Hasle H, Winther JF, Olsen JH, Olsen J. Do parents of children with congenital malformations have a higher cancer risk? A nationwide study in Denmark. British journal of cancer. 2002;87(5):524-8.

6. Merks JH, Ozgen HM, Koster J, Zwinderman AH, Caron HN, Hennekam RC. Prevalence and patterns of morphological abnormalities in patients with childhood cancer. JAMA : the journal of the American Medical Association. 2008;299(1):61-9.

7. Loeb LA, Springgate CF, Battula N. Errors in DNA replication as a basis of malignant changes. Cancer research. 1974;34(9):2311-21.

8. Loeb LA. Human cancers express mutator phenotypes: origin, consequences and targeting. Nature reviews Cancer. 2011;11(6):450-7.

9. Cervelli T1, Borghini A, Galli A, Andreassi MG. DNA damage and repair in atherosclerosis: current insights and future perspectives. Int J Mol Sci. 2012;13(12):16929-44.

10. Mehes K, Kosztolanyi G. Clinical manifestations of genetic instability overlap one another. Pathology oncology research : POR. 2004;10(1):12-6.

11. Chan D, Delbès G, Landry M, Robaire B, Trasler JM. Epigenetic alterations in sperm DNA associated with testicular cancer treatment. Toxicol Sci. 2012 Feb;125(2):532-43
