# Supplementary material for: Cancer therapy and risk of congenital malformations in children fathered by men treated for testicular germ-cell cancer: A nationwide register study
Source: PLoS Med. 2019 Jun 4;16(6):e1002816. doi: 10.1371/journal.pmed.1002816 (PMC6548355; doi:10.1371/journal.pmed.1002816)
Supplement: S3 Text — (DOCX) [file pmed.1002816.s013.docx]

**The impact of testicular cancer and its treatment on the health of the offspring**

**Study coordinator:**

Aleksander Giwercman, Lund University: [aleksander.giwercman@med.lu.se](mailto:aleksander.giwercman@med.lu.se)

**Other investigators**

***From Denmark:***

Gedske Daugaard: [gedske.daugaard@regionh.dk](mailto:gedske.daugaard@regionh.dk)

Mikkel Bandak: [mikkel.bandak@regionh.dk](mailto:mikkel.bandak@regionh.dk)

Allan Jensen: [allan@cancer.dk](mailto:allan@cancer.dk)

***From Norway:***

Hege S. Haugnes: [hege.sagstuen.haugnes@uit.no](mailto:hege.sagstuen.haugnes@uit.no)

Tom Wilsgaard: [tom.wilsgaard@uit.no](mailto:tom.wilsgaard@uit.no)

Torgrim Tandstad: [Torgrim.Tandstad@stolav.no](mailto:Torgrim.Tandstad@stolav.no)

Tone Bjørge: [tone.bjorge@uib.no](mailto:tone.bjorge@uib.no)

***From Sweden***

Olof Ståhl: [olof.stahl@med.lu.se](mailto:olof.stahl@med.lu.se)

Lars Rylander: [lars.rylander@med.lu.se](mailto:lars.rylander@med.lu.se)

Gabriella Cohn-Cedermark: [gabriella.cohn-cedermark@karolinska.se](mailto:gabriella.cohn-cedermark@karolinska.se)

Carina B. Nord: carina.berglund-nord@karolinska.se

**Background**

In a study based on a database of almost 1.8 million singleton children born in Denmark and Sweden during the period 1994-2005, we found that 8,670 of the children had fathers who previously had been diagnosed with cancer. These data has been generated by linking the information from several health registries. The risk for major congenital malformations was significantly increased (3.7% vs. 3.2%; Relative Risk = 1.17, 95% CI 1.05-1.31, p=0.004) in offspring of male cancer survivors as compared to the background population (1).

In this registry-based study, no information on cancer treatment was available. However, whereas a paternal history of hematological malignancy, generally treated with chemotherapy, was associated with an increased risk for congenital abnormalities, although not statistically significant, no increased risk was seen among children with a paternal history of testicular seminoma of whom the majority were treated with radiotherapy (stage I disease). In contrast, a paternal history of skin cancer, for which the standard treatment is surgical excision, was accompanied by almost 40% increased risk of congenital abnormalities. These findings suggest that the increased risk of congenital abnormalities may be related more to the paternal disease per se than to its treatment.

Biologically, genomic instability (GI) might be the possible link between both the paternal cancer and the risk for malformations among the offspring since increased GI leads to the initiation of cancer and could, via the same abnormalities in the DNA of the paternal spermatozoa, be transmitted to the child. In support of this hypothesis is the finding that paternal grandfathers and grandmothers with non-familial achondroplasia (common form of dwarfism) had significantly more cancers than maternal grandfathers and grandmothers (2). Notably, all sporadic achondroplasia cases had inherited the mutation from their father (3) with a strong link to increased paternal age (4). Also parents of children born with cleft lip/palate have an increased cancer risk (5). Finally, children with cancer have an overall increased risk of minor malformations, besides the classical cancer-predisposing syndromes (6).

Many years ago, Loeb et al proposed and developed the hypothesis that cancer cells exhibit a mutator phenotype (7). The basic premise is that normal mutation rates are insufficient to account for the multiple mutations observed in cancer cells and therefore mutations in critical genes, which have the ability to increase mutation rates, are essential to account for the large number of aberrations observed in human tumours. The mutator phenotype arising from those genes can have diverse manifestations, such as point mutations, microsatellite instability and loss of heterozygosity. The mutator hypothesis postulates that an initial mutation generates further mutations, including those in additional genetic stability genes, resulting in a cascade of mutations throughout the genome (8). A low-grade constitutional mutator phenotype that is present in persons predisposed to cancer, could also involve germ cells and manifest itself as mutations leading to malformations.

Another possible mechanism is an increase in the mutation rate during meiosis leading to genetic aberrations in the spermatozoa, which subsequently are transmitted to the offspring (9).

However, GI has not only been linked to cancer and congenital malformations, but also to pre-mature aging and to an increased risk of diseases associated with aging as e.g. diabetes and atherosclerosis (10). Thus, one can speculate that an increased level of GI can lead to a diversity of morbidities which can manifest already in childhood or early adulthood.

Both irradiation and chemotherapy might induce an increased level of GI in germ cells (11) and thereby contribute to an elevated risk of congenital malformations as well as morbidity in offspring of men treated for testicular cancer.

Another mechanism of action linking paternal cancer and its treatment to increased morbidity in the offspring, is induction of epigenetic changes by cancer therapy (12)

Germ-cell testicular cancer (TC) mainly affects young males typically aged 20-40 years. Norway and Denmark have the highest incidence of TC in the world, and the incidence is increasing (13). Fortunately, the treatment of germ-cell TC represents a medical success story with today's 5-year survival rates exceeding 95% (14). The high cure rate has been achieved by several factors of which the introduction of cisplatin for the treatment of metastatic disease from the late 1970s is the most important (15, 16). A vast proportion of the TC men have not yet fathered any child at the time when the cancer diagnosis is made. Unfortunately, both the cancer *per se* and its treatment may have a negative effect on their fertility, why use of assisted reproduction may become necessary.

**Scientific questions:**

1. Offspring of TC patients:
   1. Are TC and/or its treatment associated with generally increased risks of morbidities among the offspring (i.e. hospitalisation, specific diseases like diabetes, asthma/allergy, cardiovascular disease and different autoimmune diseases)?
   2. Are there increased risks of congenital malformations and/or other perinatal abnormalities in the offspring?
   3. Are TC and/or its treatment associated with an increased risk of cancers in the offspring?
2. What is the success rate and the risk of miscarriage for TC patients using assisted reproductive technology?

The study will be based on the joint Danish-Norwegian-Swedish database of approximately 15 000 TC patients. By linking this database to the available national registries we will try to answer the questions defined above.

Calculations should be made for:

1. All children of men with a diagnosis of testicular cancer
2. Pre- vs. post- cancer treatment births
3. Natural conception vs. IVF treatments
4. Different histologies (seminoma vs. non-seminoma) and treatment modalities (surgery, radiotherapy, chemotherapy) for testicular cancer

The findings should, when possible, be related to what treatment the fathers have received.

If possible – for post-cancer treatment births, use of cryopreserved (OBS. donated sperms) vs. fresh spermatozoa should be taken into consideration.

***Data sources***

*Patient databases:*

Unique databases of TC patients, established in Denmark, Norway and Sweden (see below) will be utilized. Patients who died within two years from diagnosis, or those with a previous cancer diagnosis according to the national Cancer Registries (except previous non-melanoma skin cancer) will be excluded. All registry information will be linked based on the personal identification number.

Denmark

The DaTeCa (Danish Testicular Cancer) database is a scientific database constituting approximately 6000 Danish male patients with germ-cell cancer (GCC) diagnosed between 1 January 1984 and 31 December 2007. The database includes cancers of gonadal and extragonadal origin.

Patients have been identified through the Danish Cancer Registry and hospital files. The Danish Cancer Registry houses information on all cancer patients in the Danish population, and the diagnoses has been confirmed through manual review of pathology reports. The database contains detailed information on more than 300 variables with relation to stage, treatment, relapses, pathology, tumor markers, kidney function, lung function, etc. Data has been collected from medical files and pathology reports. Cause of death has been collected from the Danish Registry of Causes of Death and cross-checked against pathology reports. By merging with national registries, complete clinical information has been obtained, and vital status is available until the time of analysis. Electronic questionnaires has been send out to all patients alive and this questionnaire includes question concerning fertility and children.

Treatment of TC in Denmark has over the years been organized through a national disease-specific group and the treatment has been aligned between the hospitals and with international guidelines. A common treatment strategy and follow-up has been performed across the centers. Surveillance has been standard treatment for stage I patients, radiotherapy for stage IIa and IIb seminoma and other patients with disseminated disease has been treated with 3 or 4 cycles of bleomycin-etoposide-cisplatin (BEP).

Treatment of TC in Denmark has over the years been increasingly centralized and is now only treated at three university hospitals. Today, all stages of TC are treated in these three departments. Guidelines for treatment are written down in national guidelines (www.ducg.dk) described by a multidisciplinary group with representation from all attending departments and other relevant specialties in the treatment of testicular cancer.

A prospective database related to the Danish Multidisciplinary Cancer Group for testicular cancer has been started in 2013.

Sweden

TC patients will be identified from the Swedish part of the clinical SWENOTECA (Swedish Norwegian Testicular Cancer Group) database. The SWENOTECA registry holds information on clinical stage [CS I-IV and tumor marker positive disease (Mk+)] , treatment modality and relapse for up to 10 years after diagnosis, for non-seminomas patients since 1995 and seminoma patients since 2000. All Swedish cancer centers report to the SWENOTECA database, which is then cross-checked with the Swedish Cancer Registry once a year through the personal national registration numbers assigned to each resident in Sweden at birth or permanent residency. The completeness of the registries is almost 100%. A total of 2292 TCSs with unilateral TC at ages 18–60 years were diagnosed from 1 July 1995 (seminomas from 1 July 2000) to 31 December 2007.

Norway

We are currently in the process of establishing a complete Norwegian database consisting of all men diagnosed with TC from January 1^st^ 1980 until December 31^st^ 2009. These men will be identified through databases at each university hospital cross-checked with data from the National Cancer Registry, thus ensuring completeness of the database. The cases are in part already registered within the SWENOTECA database, or registered as part of other previous or ongoing research projects. For men not already registered in a database, clinical parameters will be registered from the medical records. The establishment of this database has been approved by the Regional Ethical Committee. We expect that between 5000 and 6000 men has been treated for TC during the time period 1980-2009.

***Registries to be utilised***

Denmark

*Civil Registration System:* Updated daily and includes all residents of Denmark, their civil status, links to their parents, and a unique personal identification number, allowing linkage of information from population-based health registries.

*Medical Birth Registry:* Established in 1973 and contains information on all live and stillbirths in Denmark.

*Cancer Registry:* Records information on cancer patients in Denmark since 1943, including information on site of tumor (ICD-7 codes), histological type (ICD-O codes), with completeness shown to be 95%–98%.

*Hospital Discharge Registry:* Contains discharge diagnoses (up to 20) for all hospitalizations (from 1977) and outpatient visits (from 1995) in Denmark.

*The Danish National Prescription Register:* Contains information regarding all pharmaceuticals prescribed in Denmark by physicians since 1995. The prescription is linked to the personal number of the subject to whom the medicine was prescribed.

*IVF registry:* Includes data on all IVF treatments in Denmark performed since 1994. Since it does not contain any information on use of fresh vs. cryopreserved (from the patient or a donor) semen, such data need to be retrieved directly from the involved fertility clinics.

Sweden

*Total Population Registry:* The civil registration of the inhabitants of Sweden, assigning all a unique personal identification number.

*Multigenerational Registry:* Contains information on first-degree relatives of all Swedish citizens born after 1931 and still alive in 1961, or born in 1961 or later, with close to 100% coverage.

*Medical Birth Registry:* Covers nearly all (>98%) children born in Sweden. Between 1993 and 2005, IVF and ICSI treatments were given at 18 public or private clinics, with data on all treatments leading to delivery of a baby reported to the Swedish National Board of Health, and thereby to the registry.

*Cancer Registry:* Mandatory reporting of cancers in Sweden since 1958, with agreement between clinical and cytological or histological diagnoses close to 100% coverage. Information includes site of tumour (ICD-7 codes), histological type (ICD-O/2 codes for 1994-2004 and ICD-O/3 codes for 2005), and basis of and date of diagnosis.

Congenital malformations: Information retrieved from the Medical Birth Registry, and supplemented with data from the *Swedish Registry of Congenital Malformations*, and the Hospital Discharge Registry.

*National patient registry (NPR):* From 1987 NPR includes all in-patient care in Sweden. NPR includes 50 million discharges for the period 1964 to 2006. The registry contains, from 2001, also outpatient visits including day surgery and psychiatric from both private and public caregivers. Primary care is not yet covered in the NPR.

Pharmaceutical directory registry (Läkemedelsregistret): Contains information regarding all pharmaceuticals prescribed in Sweden by physicians since 1999. The prescription is linked to the personal number of the subject to whom the medicine was prescribed.

*IVF registry:* Includes data on all IVF treatments in Sweden performed since 2007.

Norway

*National Population Registry:* The civil registration of the inhabitants of Norway, assigning all a unique personal identification number, allowing linkage of information from population-based health registries.

*Cancer Registry of Norway:* Has registryed information on cancer patients in Norway since 1951, including information on site of tumor (ICD-7 codes) and histological type (ICD-O codes), with a high degree of completeness.

*Medical Birth Registry:* A national birth registry with detailed registration of all births in Norway since 1967. Information regarding birth complications, stillbirths, congenital malformations as well as information regarding assisted reproduction is registryed. Data regarding assisted reproduction has been registryed since 1984.

*The Norwegian Prescription Database:* Contains information regarding all pharmaceuticals prescribed by physicians in Norway since 2004. The prescription is linked to the personal number of the subject to whom the medicine was prescribed.

*Norwegian Patient Registry (NPR):* Includes information about all patients who have been treated in the secondary and tertiary health care since 2008, both out-patient visits and hospitalisations.

***Study cohorts***

We will investigate the associations between TC as well as its treatment and the end points defined under Scientific Questions (above). In a cohort design, 4 unexposed children per exposed child will be randomly selected matched on date of birth.

***Exposures***

Paternal history of diagnosis and type of treatment for TC and whether pregnancy was achieved naturally (spontaneously or via non-IVF/ICSI fertility treatments) or through IVF/ICSI constitute the exposures of interest for the proposed cohort study.

***Outcomes***

The outcomes of interest include 1) Success rate and the risk of miscarriage for TC patients using assisted reproductive technology, 2) cancer and other morbidity among the offspring (i.e. number of hospitalizations and specific somatic diseases like diabetes, asthma/allergy, cardiovascular disease and autoimmune diseases) and 3) congenital malformations and/or other perinatal abnormalities among the offspring.

***Potential confounders***

Potential confounders will be selected and applied based on the literature, and their availability in the relevant Danish, Norwegian and Swedish population-based health registries. Potential confounders includes calendar time, age at first male infertility diagnosis, type of assisted reproductive technology (IVF and/or ICSI), if any, number of children, place of residence, maternal smoking and socioeconomic status (level of education and disposable income).

***Statistical analyses***

For outcomes not ascertained at birth, the cohort members will be followed from birth until the first of the following events: 1) event of interest; 2) death; 3) emigration; 4) designated “missing person” in the Civil Registration System; or 5) end of follow-up (December 31, 2015).

Two different kinds of analytic approaches will be used:

For outcomes ascertained at birth (success rate, risk of miscarriage, congenital malformations and other perinatal abnormalities) logistic regression models will be applied.

For cancer, number of hospitalizations and specific somatic diseases among the children, a Cox regression model will be used (conditions with follow-up time).

All analyses will be performed using SAS and R statistical software.

***Power analysis***

The project will, in most cases, have sufficient statistical power, which can be illustrated by the following example. If we assume that half of the men with a history of TC will become fathers and if we further assume that 3.5 % of the children from the general population will have a low birth weight (<2500 g), will we with 80% statistical power have the possibility to observe a significant (p<0.05) increased risk of 1.18 or higher.

***Ethical considerations***

Ethical committees in Denmark, Norway and Sweden will be asked for permission to perform the study. Depending on the decision of the ethical committee the patients will be informed about the study by:

1. Announcement in the newspapers (opt out), or by
2. Letter (opt out or opt in)

In Denmark an ethical permission for this patient cohort is already obtained, but in order to cover all registries to be utilised, an amendment will be necessary.

If the study shows no health risk for the offspring of TC patients, this information can be used to reassure these men that their disease implies no hazard for their children.

In case of any link between the disease or cancer treatment and any of the outcomes to be included in this study is shown, this information will also be of value in counselling the patients, i.e. as considers use of fresh or cryopreserved spermatozoa, as well as in development of preventive measures in relation to the disease risk in their children. Our findings may also become a “model” for future management of the offspring of cancer survivors.

Use of cancer registries gives us the possibility to answer some important clinically and biologically relevant questions without directly getting in contact with the patients and their children. Thereby creating an unnecessary worry among those subjects can be avoided.

An application to the national data protection agencies will be ssubmitted.

***Cancer relevance***

An increasing number of young cancer patients are being cured. For that reason the aspects of their reproductive function, which may be impaired by the cancer disease *per se* as well as by its treatment is of outermost importance.

Whereas we now have a large amount of data concerning the impact of TC and its treatment on fertility of the patients, very little is known about the implications for the health of the offspring.

As indicated above (see under “Ethical considerations”) our findings will have important implications for counselling of young male cancer survivors.

If we find a link between cancer *per se* and adverse health outcome among the children, this will provide us important information about the biology of the cancer disease.

***Timeline***

The project will cover a total period of 36 months, starting 01.01.2016 and ending 31.12.2018:

Phase 1: 01.01.2016-31.12.2016: Obtain data from registries.

Phase 2: 01.01.2017-31.10.2017: Data linkage, data cleaning and data harmonization.

Phase 3: 01.11.2017-30.06.2018: Data analysis.

Phase 4: 01.07.2018-31.12.2018: Prepare manuscripts.

As per August 20^th^ 2016, the project group is following the time schedule indicated above. Thus, provided continuous funding of the project, it should be finalized within the timeline indicated above.

***National/international collaborations***

The investigators involved in this study represent three Nordic countries – Denmark, Norway and Sweden. All of them have extensive collaboration with other groups within this area of cancer research – nationally and internationally.

**References**

1. Stahl O, Boyd HA, Giwercman A, Lindholm M, Jensen A, Kjaer SK, et al. Risk of birth abnormalities in the offspring of men with a history of cancer: a cohort study using Danish and Swedish national registries. Journal of the National Cancer Institute. 2011;103(5):398-406.

2. Stoll C, Feingold J. Do parents and grandparents of patients with achondroplasia have a higher cancer risk? American journal of medical genetics Part A. 2004;130A(2):165-8.

3. Wilkin DJ, Szabo JK, Cameron R, Henderson S, Bellus GA, Mack ML, et al. Mutations in fibroblast growth-factor receptor 3 in sporadic cases of achondroplasia occur exclusively on the paternally derived chromosome. American journal of human genetics. 1998;63(3):711-6.

4. Risch N, Reich EW, Wishnick MM, McCarthy JG. Spontaneous mutation and parental age in humans. American journal of human genetics. 1987;41(2):218-48.

5. Zhu JL, Basso O, Hasle H, Winther JF, Olsen JH, Olsen J. Do parents of children with congenital malformations have a higher cancer risk? A nationwide study in Denmark. British journal of cancer. 2002;87(5):524-8.

6. Merks JH, Ozgen HM, Koster J, Zwinderman AH, Caron HN, Hennekam RC. Prevalence and patterns of morphological abnormalities in patients with childhood cancer. JAMA : the journal of the American Medical Association. 2008;299(1):61-9.

7. Loeb LA, Springgate CF, Battula N. Errors in DNA replication as a basis of malignant changes. Cancer research. 1974;34(9):2311-21.

8. Loeb LA. Human cancers express mutator phenotypes: origin, consequences and targeting. Nature reviews Cancer. 2011;11(6):450-7.

9. Cervelli T1, Borghini A, Galli A, Andreassi MG. DNA damage and repair in atherosclerosis: current insights and future perspectives. Int J Mol Sci. 2012;13(12):16929-44.

10. Mehes K, Kosztolanyi G. Clinical manifestations of genetic instability overlap one another. Pathology oncology research : POR. 2004;10(1):12-6.

11. Greco M1, D'Alò F, Scardocci A, Criscuolo M, Fabiani E, Guidi F, Di Ruscio A, Migliara G, Pagano L, Fianchi L, Chiusolo P, Hohaus S, Leone G, Voso MT. Promoter methylation of DAPK1, E-cadherin and thrombospondin-1 in de novo and therapy-related myeloid neoplasms. Blood Cells Mol Dis. 2010;45(3):181-5.

12. Chan D, Delbès G, Landry M, Robaire B, Trasler JM. Epigenetic alterations in sperm DNA associated with testicular cancer treatment. Toxicol Sci. 2012 Feb;125(2):532-43

13. Larsen IK, Larønningen S, Johannesen T et al. Cancer in Norway 2012 - Cancer incidence, mortality, survival and prevalence in Norway. Cancer Registry of Norway 2014, Oslo.

Available at www.kreftregisteret.no, last accesssed August 5th 2014.

14. Verdecchia A, Francisci S, Brenner H et al. Recent cancer survival in Europe: a 2000-02

period analysis of EUROCARE-4 data. Lancet Oncol 2007; 8: 784-796.

15. Feldman DR, Bosl GJ, Sheinfeld J, Motzer RJ. Medical treatment of advanced testicular

cancer. JAMA 2008; 299: 672-684.

16. Einhorn LH, Donohue J. Cis-diamminedichloroplatinum, vinblastine, and bleomycin

combination chemotherapy in disseminated testicular cancer. Ann Intern Med 1977; 87: 293-298.
